# Filling in the GAPS: evaluating completeness and coverage of open‐access biodiversity databases in the United States

**DOI:** 10.1002/ece3.2225

**Published:** 2016-06-12

**Authors:** Matthew J. Troia, Ryan A. McManamay

**Affiliations:** ^1^ Environmental Sciences Division Oak Ridge National Laboratory Oak Ridge Tennessee 7831

**Keywords:** Biodiversity, Global Biodiversity Information Facility, museum collections, National Rivers and Streams Assessment, National Water Quality Assessment, North American Breeding Bird Survey, Regional Environmental Monitoring and Assessment Program, species distribution modeling, Wallacean shortfall

## Abstract

Primary biodiversity data constitute observations of particular species at given points in time and space. Open‐access electronic databases provide unprecedented access to these data, but their usefulness in characterizing species distributions and patterns in biodiversity depend on how complete species inventories are at a given survey location and how uniformly distributed survey locations are along dimensions of time, space, and environment. Our aim was to compare completeness and coverage among three open‐access databases representing ten taxonomic groups (amphibians, birds, freshwater bivalves, crayfish, freshwater fish, fungi, insects, mammals, plants, and reptiles) in the contiguous United States. We compiled occurrence records from the Global Biodiversity Information Facility (GBIF), the North American Breeding Bird Survey (BBS), and federally administered fish surveys (FFS). We aggregated occurrence records by 0.1° × 0.1° grid cells and computed three completeness metrics to classify each grid cell as well‐surveyed or not. Next, we compared frequency distributions of surveyed grid cells to background environmental conditions in a GIS and performed Kolmogorov–Smirnov tests to quantify coverage through time, along two spatial gradients, and along eight environmental gradients. The three databases contributed >13.6 million reliable occurrence records distributed among >190,000 grid cells. The percent of well‐surveyed grid cells was substantially lower for GBIF (5.2%) than for systematic surveys (BBS and FFS; 82.5%). Still, the large number of GBIF occurrence records produced at least 250 well‐surveyed grid cells for six of nine taxonomic groups. Coverages of systematic surveys were less biased across spatial and environmental dimensions but were more biased in temporal coverage compared to GBIF data. GBIF coverages also varied among taxonomic groups, consistent with commonly recognized geographic, environmental, and institutional sampling biases. This comprehensive assessment of biodiversity data across the contiguous United States provides a prioritization scheme to fill in the gaps by contributing existing occurrence records to the public domain and planning future surveys.

## Introduction

There is increasing recognition that ecological and evolutionary processes operate in response to natural and anthropogenic factors that are apparent at regional, continental, and even global scales. Research in the fields of macroecology and landscape ecology has demonstrated that broad‐scale environmental variation and spatial processes play important roles in generating and maintaining biodiversity (Brown 1995; Turner et al. 2001). Multiple contemporary threats to biodiversity are apparent at similarly broad spatial scales, including habitat loss and fragmentation stemming from the alteration of natural landscapes, climate change, and intercontinental faunal and floral exchanges (Rahel 2000; Bates et al. 2008; Newbold et al. 2015). In freshwaters, additional broad‐scale threats to biodiversity include eutrophication and hydrologic alteration (Bennett et al. 2001; Poff et al. 2007; Esselman et al. 2011).

Primary biodiversity data – observations of particular species at given points in time and space – are essential to understanding how these broad‐scale processes affect the distribution of species and biodiversity across the globe (Soberón and Peterson 2004; Peterson et al. 2010). A major challenge that remains, however, is inadequate primary biodiversity data for many regions and taxonomic groups throughout the world. Recent efforts to overcome this Wallacean shortfall have sought to compile species occurrence records using open‐access database platforms (Lomolino and Lawrence 2004; Whittaker et al. 2005). For example, the Global Biodiversity Information Facility (GBIF) currently provides online open access to over 521 million occurrence records representing more than 1.4 million species (Edwards et al. 2000; Yesson et al. 2007). These databases have allowed investigators to test ecological and evolutionary hypotheses that explain the natural generation and maintenance of biodiversity as well as document contemporary and human‐induced changes in biodiversity (*e.g*., Rahel 2000; Kozak and Wiens 2006; Mitchell and Knouft 2009). Moreover, recent developments in GIS software, broad‐scale environmental data layers (*e.g*., Worldclim; Hijmans et al. 2005), and refinement of statistical techniques (*e.g*., MaxEnt; Phillips et al. 2006) have led to the extensive use of these open‐access databases in species distribution modeling (SDM; Guisan and Thuiller 2005; Broennimann et al. 2007; Domínguez‐Domínguez et al. 2006) and biodiversity mapping (Sousa‐Baena et al. 2013; García‐Roselló et al. 2015).

Broad‐scale databases describing species occurrences, such as GBIF, are frequently composed of many smaller (*i.e*., narrower spatial extent or fewer records) surveys of multispecies assemblages or single‐species occurrence records (*e.g*., georeferenced museum vouchers) collected for many different purposes and by many different scientists, natural resource managers, and even recreational naturalists. This data compilation scheme frequently results in incomplete inventories of the species occupying a survey location and inadequate survey coverage along three important ecological dimensions: time, space, and environment (Ladle and Hortal 2013). Survey *completeness* is important for biodiversity studies that seek to statistically model and map patterns in species richness (Lobo 2008; Chao and Jost 2012). Indeed, survey completeness is an overriding factor affecting observed richness for a given survey location (Hortal et al. 2007; Soberón et al. 2007). A variety of analytic approaches have been developed to quantify the completeness of biodiversity surveys (reviewed by Colwell et al., 1994). Many of these approaches use parametric or nonparametric algorithms to estimate “expected” (*i.e*., actual) species richness based on the frequency of individual species occurrences within a survey location. The proportion of observed richness versus expected richness is then computed and used as a metric of survey completeness (Hortal et al. 2006; Soberón et al. 2007). An alternative approach characterizes the final (*i.e*., right side) slope of the species accumulation curve for a given survey location. Slopes near zero suggest that richness has reached an asymptote with the currently available number of occurrence records and is indicative of high completeness (Yang et al. 2013).

Regarding survey *coverage*, different ecological and evolutionary questions require consistent data coverage along one or more dimensions of time, space, and environment (Rahel 2000; Broennimann et al. 2007; Pearman et al. 2008). Uneven representation of key environmental gradients by occurrence records can strongly influence the accuracy of SDMs and the perceived importance of environmental predictor variables used to build those SDMs (Kadmon et al. 2004; Loiselle et al. 2008; Tessarolo et al. 2014). Similarly, uneven representation of spatial and environmental gradients also affects the performance of modeling efforts aimed at predicting and mapping patterns in biodiversity (*e.g*., species richness) across unsurveyed regions (Dennis and Thomas 2000; Ladle and Hortal 2013). Discrepancies in environmental data coverage between two regions (*i.e*., incomplete space‐by‐environment data coverage) can influence model transferability, which can weaken inferences made about geographic range limits and niche shifts of invasive species or tests of local adaptation among geographically separated populations (Broennimann et al. 2007; Peterson et al. 2007). Spatial data gaps through time (*i.e*., space‐by‐time) can influence the ability to detect geographic range shifts over time (Tingley and Beissinger 2009) or biotic homogenization between regions (Rahel 2000). Gaps in data along environmental gradients and through time (*i.e*., environment‐by‐time) can limit the detection of environmental niche evolution – a process that has important implications for understanding natural species richness patterns or predicting species adaptive potential in the face of human‐induced global change (Pearman et al. 2008). As with completeness, a variety of analytic approaches have been developed to quantify the coverage of biodiversity surveys. These include direct measurement of the frequency distributions of biodiversity surveys along key environmental gradients (*e.g*., Kadmon et al. 2003; Loiselle et al. 2008) as well as summarizing environmental variation among survey locations as a surrogate of biodiversity (*e.g*., Hortal and Lobo 2005).

The aim of this study was to compare completeness and coverage among three open‐access databases representing ten taxonomically diverse groups of macro‐organisms in the contiguous United States (amphibians, birds, freshwater bivalves, crayfish, freshwater fish, fungi, insects, mammals, plants, and reptiles). First, by comparing completeness among a database composed of many smaller data compilation efforts (GBIF) with databases of systematic survey efforts (North American Breeding Bird Survey, federally administered fish surveys), our goal was to assess the utility of data compilation efforts with regard to describing spatial variation in species richness. Second, we characterized the coverage of biodiversity surveys derived from these databases along two spatial gradients (latitude and longitude); three natural environmental gradients (elevation, mean annual temperature, and mean annual precipitation); three anthropogenic environmental gradients (urban land cover, agricultural land cover, and total disturbed land cover); and two gradients of future climate change (forecasted change in mean annual temperature and mean annual precipitation between the late 1900s and the 2080s). Characterizing coverage along these latter two gradients of anthropogenic environmental change is an important, yet overlooked, component of biodiversity data planning. Lastly, by synthesizing completeness and coverage among multiple databases, taxonomic groups, and ecologically relevant gradients, our goal was to elucidate the causes of data gaps and offer an objective path toward comprehensive biodiversity conservation in the United States.

## Materials and Methods

### Compilation of occurrence records

We downloaded georeferenced occurrence records within the contiguous United States from GBIF. This data repository is likely the most comprehensive source of open‐access species occurrence records and includes records from other frequently used data repositories that are specific to a geographic region (*e.g*., BISON), taxonomic group (*e.g*., FishNet2, HerpNet, MANIS), or institution (*e.g*., Kansas University Biological Survey) (Yesson et al. 2007). As such, numerous studies have made use of GBIF data as presence‐only records in SDMs (*e.g*., Giovanelli et al. 2008) and biodiversity studies (*e.g*., Pineda and Lobo 2009). For the present analysis, taxonomic keywords were used to download records from GBIF for amphibians (Class: Amphibia), birds (Class: Aves), freshwater bivalves (Order: Unionoida), crayfish (Order: Decapoda), fishes (Class: Actinopterygii), fungi (Kingdom: Fungi), insects (Class: Insecta), mammals (Class: Mammalia), plants (Kingdom: Plantae), and reptiles (Class: Reptilia). Records were screened and those with (1) “no known coordinate issues”; (2) a sampling year between 1800 and 2013; (3) a taxonomic rank of “species”; and (4) a record type of “specimen” were retained for the analysis. In addition to GBIF records, we obtained georeferenced routes from the North American Breeding Bird Survey (hereafter BBS; Pardieck et al. 2014). The BBS data have been used previously in national and continental‐scale studies of avian ecology and conservation (*e.g*., Bahn and McGill 2007; Peterson et al. 2007). We also obtained georeferenced federal fish surveys (hereafter FFS) from the Environmental Protection Agency's Regional Environmental Monitoring and Assessment Program and National Rivers and Streams Assessment as well as the United States Geological Survey's National Water Quality Assessment. These three sources of fish distributional data have been collated previously and used as a comprehensive presence–absence dataset in national‐scale studies of freshwater biogeography, ecology, and conservation (*e.g*., Herlihy et al. 2006; Mitchell and Knouft 2009; Mims and Olden 2012). The BBS and FFS datasets provide an informative comparison with the GBIF datasets, as they represent systematic (and presumably less biased) sampling efforts by one or several collaborating authorities.

We defined an individual occurrence record as a database row that represents an individual organism collected from a known location (i.e., latitude and longitude) and at a known time (i.e., calendar year). These occurrence records were mapped in ArcMap (version 10.1; ESRI, Inc.: Redlands, CA) and assigned to one of 83,545 grid cells (0.1° by 0.1° rectangles) distributed across the contiguous United States using the spatial join tool. These dimensions correspond to cells that range in size from 80 km^2^ (11.1 km by 7.2 km) at the northernmost latitudes to 112 km^2^ (11.1 km by 10.1 km) at the southernmost latitudes. A trade‐off exists between maximizing survey resolution (*i.e*., small grid cells) while retaining an adequate number of occurrence records within each grid cell. Previous studies indicated that a 0.1° cell size provides sufficient resolution to be useful for biodiversity research (Hortal et al. 2006; Soberón et al. 2007) and a preliminary exploration of larger and smaller sizes indicated that this size retained adequate numbers of occurrence records per cell with datasets used in this study. We defined an individual survey as all occurrence records within a grid cell. Surveys were defined over three different time periods: all records between 1800 and 2013 (hereafter “complete time period”); all records between 1990 and 2013 (hereafter “contemporary time period”); and records falling within each of ten different 20‐year intervals plus a final 14‐year interval (*i.e*., 1800–1819, 1820–1839, 1840–1859, 1860–1879, 1880–1899, 1900–1919, 1920–1939, 1940–1959, 1960–1979, 1980–1999, 2000–2013).

### Survey completeness

Three completeness metrics were computed and used to classify each survey as “well‐surveyed” or “not‐well‐surveyed.” These metrics included (1) the number of records per grid cell, (2) the Chao2 completeness index, and (3) the final (*i.e*., right side) slope of species accumulation curve (Chao 1987; Yang et al. 2013). We explored low (i.e., liberal), moderate, and high (i.e., conservative) thresholds for each of these three completeness metrics based on the range of thresholds used in previous studies (Soberón et al. 2007; Sousa‐Baena et al. 2013; Yang et al. 2013). For the low threshold, well‐surveyed cells were defined as those with ≥10 occurrence records, a Chao2 completeness metric ≥0.6, and a final species accumulation curve slope of ≤0.15. For the moderate threshold, well‐surveyed cells were defined as those with ≥25 occurrence records, a Chao2 completeness metric ≥0.7, and a final species accumulation curve slope of ≤0.10. For the high threshold, well‐surveyed cells were defined as those with ≥50 occurrence records, a Chao2 completeness metric ≥0.8, and a final species accumulation curve slope of ≤0.05.

### Survey coverage

We evaluated the coverage of well‐surveyed and not‐well‐surveyed grid cells along spatial, environmental, and temporal gradients. Natural environmental variables were acquired as raster grids (30 arc‐second resolution) from the WorldClim dataset and were spatially joined to the survey grid cells in ArcMap. These variables included elevation above sea level, contemporary mean annual temperature (MAT), and contemporary mean annual precipitation (MAP). Anthropogenic environmental variables were acquired as raster grids (30 m resolution) from the 2001 National Land Cover Database (NLCD). Percent coverage of each anthropogenic land cover class was summarized within each survey grid cell. We defined urban land cover as the sum of NLCD classes 21, 22, 23, and 24; agricultural land cover as the sum of NLCD classes 81 and 82; and total disturbed land cover as the sum of urban and agricultural land cover. Lastly, forecasted changes in mean annual temperature (∆MAT) and mean annual precipitation (∆MAP) were acquired as raster grids (30 arc‐second resolution) from the Climate Wizard tool (Girvetz et al. 2009). These projections represent differences between contemporary (1961–1990) and future (2080s) MATs and MAPs based on an ensemble average of sixteen global circulation models assuming moderate carbon emissions (*i.e*., A1B scenario). These climate change variables were spatially joined to the survey grid cells using ArcMap.

We evaluated coverage along the time gradient by comparing the frequency of surveys among each of the 20‐year time intervals to a uniform frequency distribution. We use a uniform distribution to represent the ideal null expectation of equal sampling among each of the 20‐year time intervals from 1800 to 2013. For spatial and environmental gradients, however, the conditions of the background environment are not uniformly distributed within a given region (*e.g*., contiguous US). Thus, we compared the frequency distribution of surveyed grid cells to that of all 82,545 grid cells. Next, we performed Kolmogorov–Smirnov (K–S) goodness‐of‐fit tests for each dataset‐by‐taxon‐by‐gradient combination and used the test statistic (*D‐*statistic) as an index of strong or weak (low or high values, respectively) congruence between each survey dataset and the background environment (Kadmon et al. 2004; Loiselle et al. 2008). *D*‐statistics were computed for all surveyed grid cells and well‐surveyed grid cells which we defined using the moderate completeness threshold described above. To evaluate comprehensive coverage of each survey dataset, *D*‐statistics were summed for all spatial and environmental gradients. We plotted overlapping histograms of each survey dataset and the background environment to provide visual reference and detail as to the position along each gradient where congruence was strong or weak. Analyses for spatial and environmental gradients were limited to contemporary records (*i.e*., 1990–2013) because the 2001 NLCD land cover is not representative of historical land cover. All statistical analyses were carried out using the vegan (Oksanen et al. 2007) and fossil (Vavrek 2011) libraries in the R programming environment (R Core Team, 2014).

## Results

### Survey completeness

**Figure 1:**
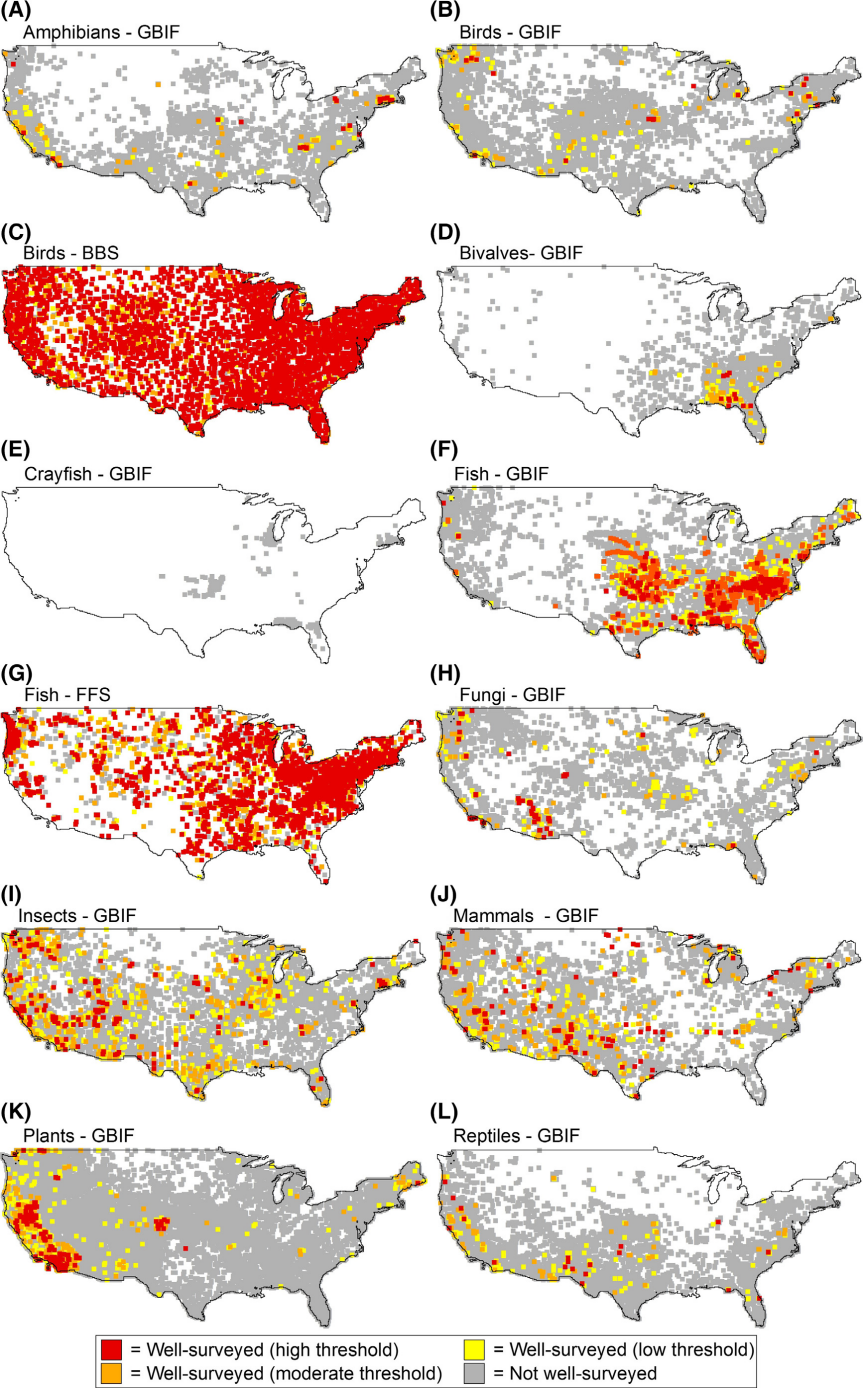
Distribution of all surveyed grid cells and well‐surveyed grid cells throughout the contiguous United States during the contemporary time period (1990–2013) derived from three open‐access biodiversity databases representing ten taxonomic groups. Note that the square symbols are enlarged (*i.e*., larger than actual grid cell area) to facilitate visualization of well‐surveyed regions.

**Figure 2:**
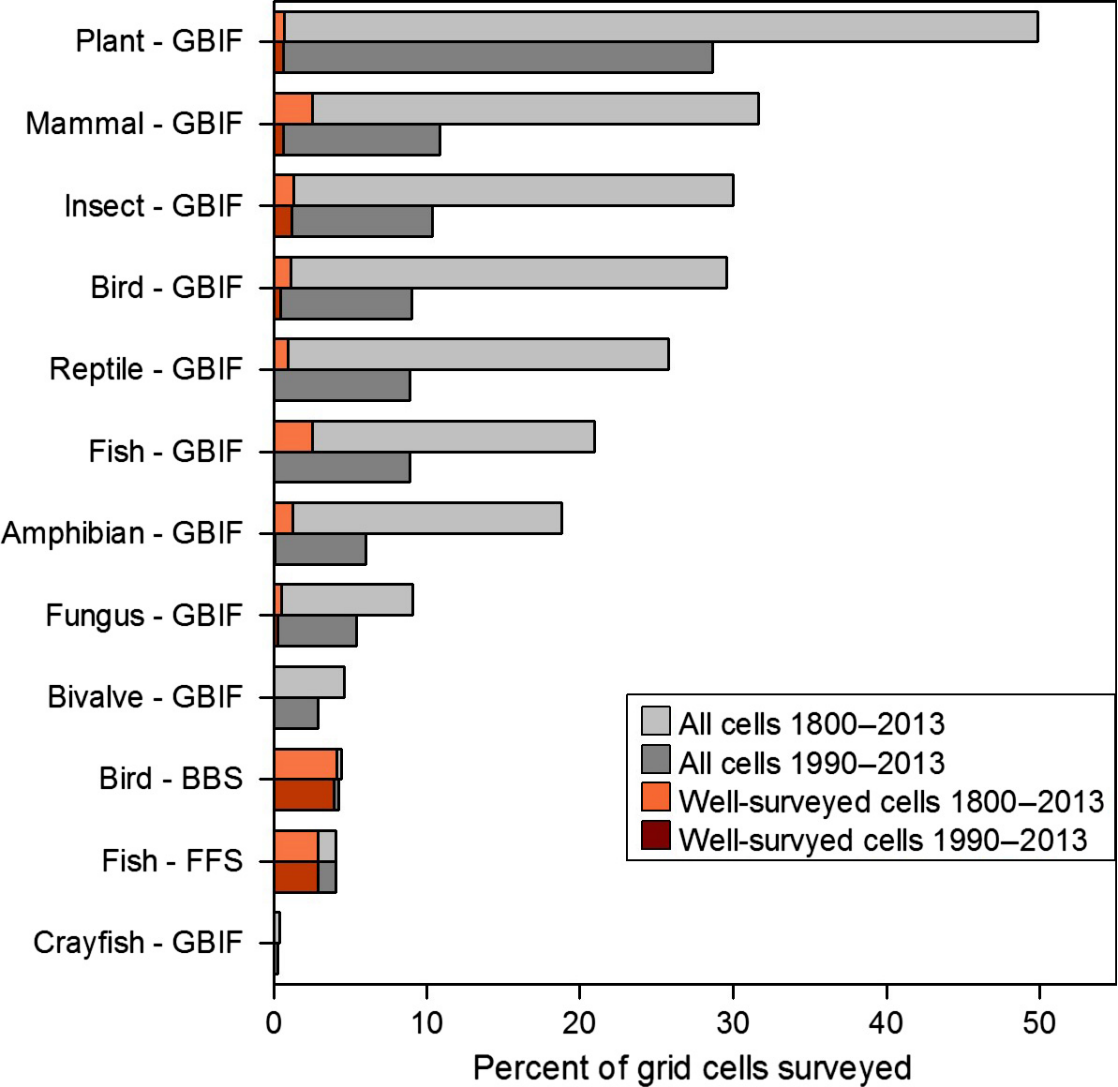
Percent of all grid cells in the contiguous United States (*N *=* *83,545) that contain surveys derived from three open‐access biodiversity databases representing the complete time period (1800–2013) and contemporary time period (1990–2013).

Our compilation of open‐access biodiversity data within the contiguous United States yielded in excess of 6.7 million GBIF records collected between 1800 and 2013, 4.8 million BBS records collected between 1963 and 2013, and 2.1 million FFS records collected between 1990 and 2008. These records were distributed among 183,165 GBIF grid cells (*i.e*., surveys), 3660 BBS grid cells, and 3,372 FFS grid cells. Since 1990, in excess of 1.9 million GBIF records, 3.0 million BBS records, and 2.1 million FFS records have been accumulated. These contemporary records were distributed among 75,836 GBIF surveys, 3523 BBS surveys, and 3372 FFS surveys (Fig. 1). For the complete time period, plant surveys from GBIF were most prevalent, followed by GBIF mammals, GBIF insects, and GBIF birds. The least prevalent surveys were GBIF crayfish, FFS fish, and BBS birds (Table 1, Fig. 2). Surveys from standardized datasets (*i.e*., BBS and FFS) were substantially more complete than those from GBIF. Specifically, 4.7% and 3.7% of GBIF‐surveyed grid cells for the complete and contemporary time periods, respectively, were classified as well‐surveyed based on the moderate or high completeness thresholds. By contrast, 82.6% and 82.3% of BBS‐ and FFS‐surveyed grid cells for the complete and contemporary time periods, respectively, were classified as well‐surveyed (Table 1, Fig. 2).

**Table 1:** Numbers of 0.1° × 0.1° grid cells surveyed across the contiguous United States. Well‐surveyed grid cells were defined using low (*i.e*., liberal), moderate, and high (*i.e*., conservative) thresholds of survey completeness metrics

Time period	Taxon	Database	Records	All surveyed grid cells (%)	Well‐surveyed grid cells (%)		
					Low	Moderate	High
Complete	Amphibian	GBIF	337,077	15,597 (19)	1713 (11)	1034 (6.6)	366 (2.3)
	Bird	GBIF	781,836	24,533 (29.9)	1914 (7.8)	923 (3.8)	246 (1)
	Bird	BBS	4,813,437	3660 (4.5)	3548 (96.9)	3414 (93.3)	2918 (79.7)
	Bivalve	GBIF	28,072	3860 (4.7)	160 (4.1)	61 (1.6)	16 (0.4)
	Crayfish	GBIF	718	316 (0.4)	0 (0)	0 (0)	0 (0)
	Fish	GBIF	609,975	17,404 (21.2)	3588 (20.6)	2121 (12.2)	686 (3.9)
	Fish	FFS	2,144,750	3372 (4.1)	2747 (81.5)	2425 (71.9)	1798 (53.3)
	Fungus	GBIF	163,803	7554 (9.2)	722 (9.6)	437 (5.8)	169 (2.2)
	Insect	GBIF	1,341,595	24,874 (30.3)	2572 (10.3)	1092 (4.4)	225 (0.9)
	Mammal	GBIF	716,183	26,240 (32)	3656 (13.9)	2089 (8)	659 (2.5)
	Plant	GBIF	2,433,827	41,401 (50.5)	1372 (3.3)	570 (1.4)	129 (0.3)
	Reptile	GBIF	347,481	21,386 (26.1)	1559 (7.3)	798 (3.7)	248 (1.2)
Contemporary	Amphibian	GBIF	44,650	4989 (6.1)	192 (3.8)	99 (2)	23 (0.5)
	Bird	GBIF	94,429	7381 (9)	202 (2.7)	84 (1.1)	19 (0.3)
	Bird	BBS	3,054,288	3523 (4.3)	3397 (96.4)	3262 (92.6)	2800 (79.5)
	Bivalve	GBIF	18,309	2407 (2.9)	123 (5.1)	53 (2.2)	11 (0.5)
	Crayfish	GBIF	610	245 (0.3)	0 (0)	0 (0)	0 (0)
	Fish	GBIF	231,249	8613 (10.5)	1692 (19.6)	971 (11.3)	242 (2.8)
	Fish	FFS	2,144,750	3372 (4.1)	2747 (81.5)	2425 (71.9)	1798 (53.3)
	Fungus	GBIF	95,118	4507 (5.5)	274 (6.1)	251 (5.6)	62 (1.4)
	Insect	GBIF	556,019	9013 (11)	1074 (11.9)	555 (6.2)	155 (1.7)
	Mammal	GBIF	128,937	7507 (9.2)	637 (8.5)	369 (4.9)	105 (1.4)
	Plant	GBIF	752,788	23,768 (29)	1042 (4.4)	540 (2.3)	174 (0.7)
	Reptile	GBIF	54,677	7406 (9)	187 (2.5)	81 (1.1)	19 (0.3)

Low threshold: ≥10 records and Chao2 completeness index ≥0.6 and final SAC slope ≤0.15.

Moderate threshold: ≥25 records and Chao2 completeness index ≥0.7 and final SAC slope ≤0.10.

High threshold: ≥50 records and Chao2 completeness index ≥0.8 and final SAC slope ≤0.05.

**Figure 3:**
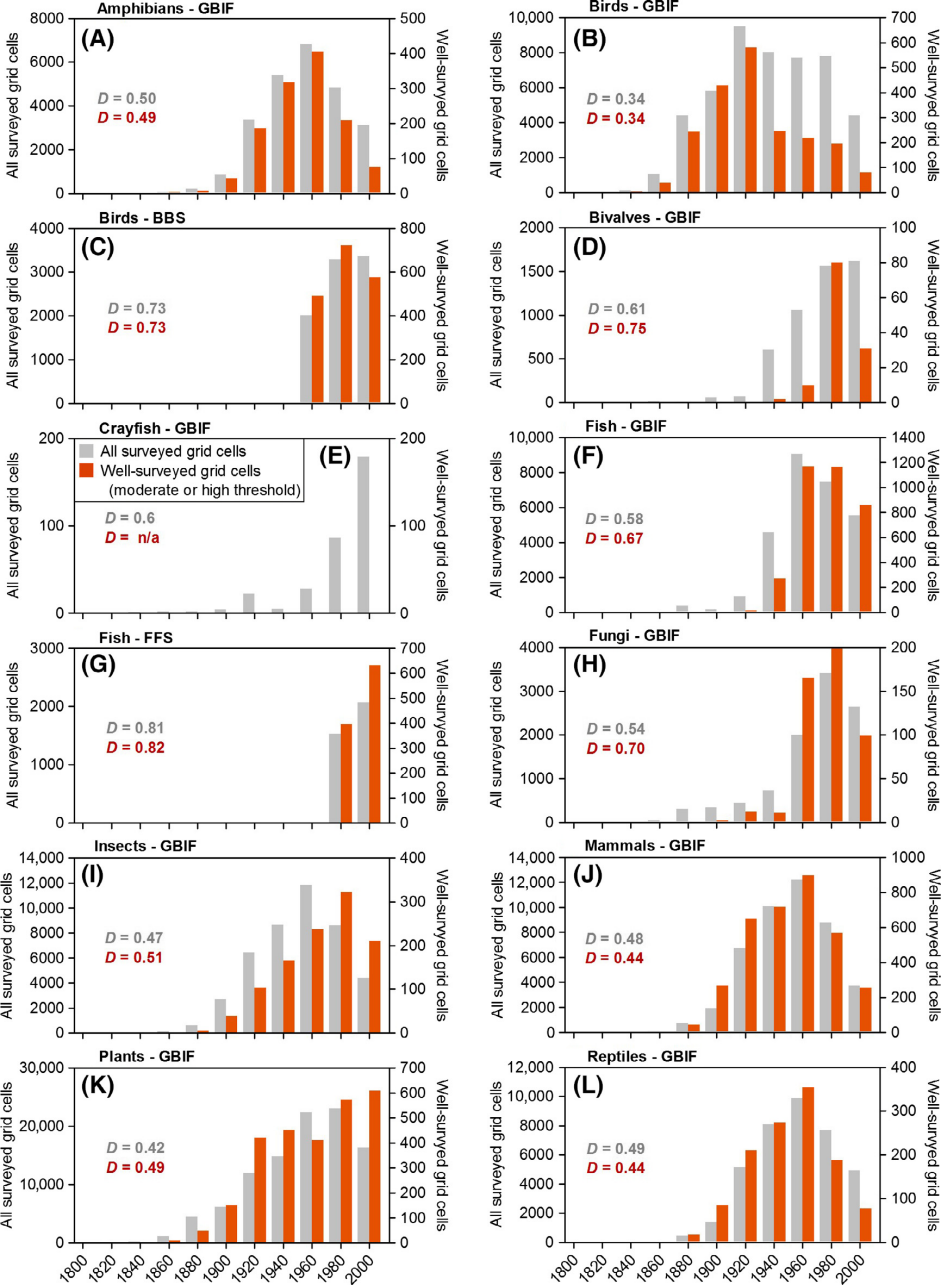
Frequency of all surveyed grid cells and well‐surveyed grid cells in each of eleven 20‐year intervals between 1800 and 2013 (most recent interval is 14 years; 2000–2013) for three open‐access biodiversity databases representing ten taxonomic groups. Note different *y*‐axis scales within and among panels.

**Figure 4:**
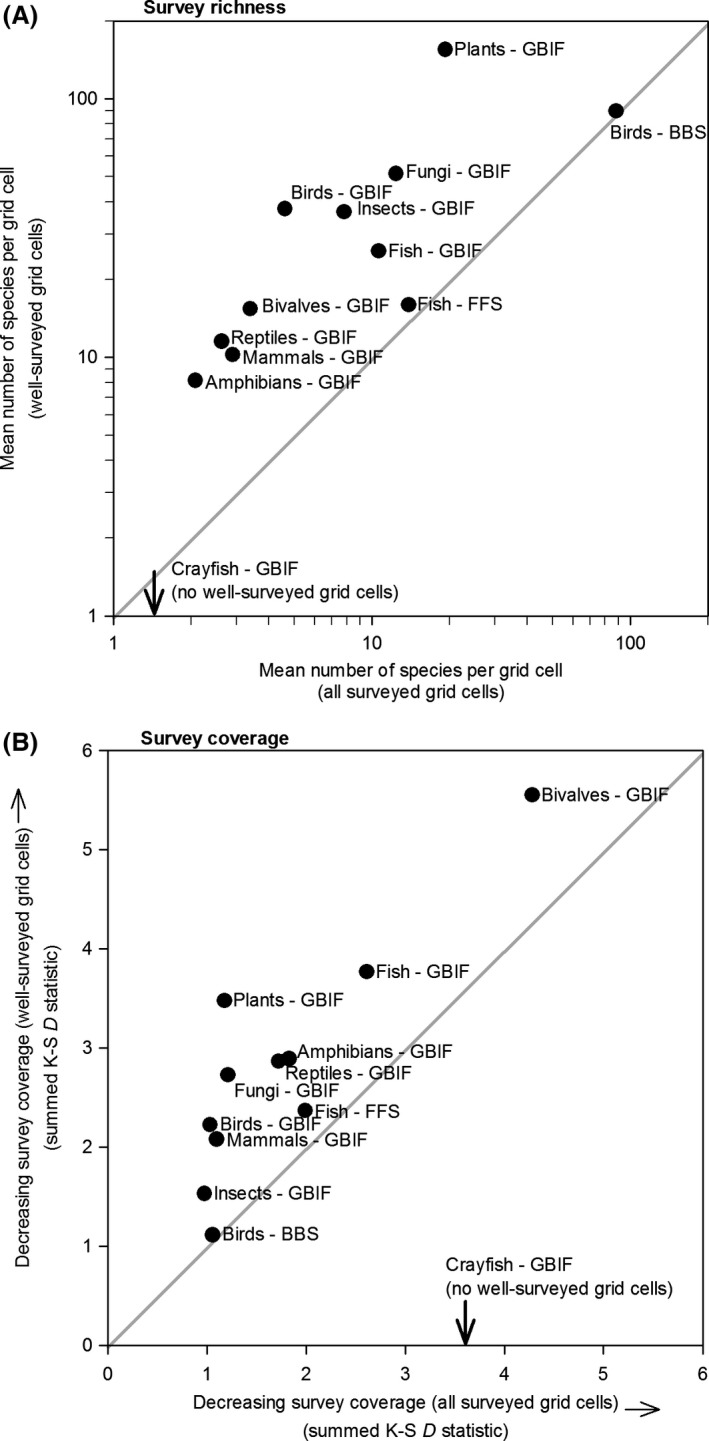
Relationship between (A) number of species per grid cell and (B) cumulative coverage of well‐surveyed grid cells versus all surveyed grid cells derived from three open‐access biodiversity databases representing ten taxonomic groups. In (B), low values represent unbiased coverage and high values represent biased coverage relative to the background environment. Note that richness and cumulative coverage could not be plotted along the *y*‐axis for crayfish because no well‐surveyed grid cells were identified.

GBIF surveys represented the longest period of record (dating back to 1800), followed by the BBS surveys (1967) and FFS surveys (1990) (Fig. 3). GBIF surveys, particularly those classified as well‐surveyed, were most prevalent since approximately 1920. Nevertheless, a substantial number of well‐surveyed grid cells were available from the nineteenth century for birds (116 grid cells), mammals (22 grid cells), and plants (9 grid cells). The average number of species inventoried per grid cell (*i.e*., survey richness) was highest for birds, plants, and fungi and lowest for crayfish, amphibians, and mammals. For most taxa, well‐surveyed grid cells contained more species (upper left diagonal in Fig. 4A) than did all (*i.e*., both well‐surveyed and not‐well‐surveyed) surveyed grid cells. Two exceptions were BBS birds and FFS fish, both of which contained similar numbers of species for well‐surveyed grid cells and all grid cells (1:1 line in Fig. 4A).

### Survey coverage

Coverage indices (*i.e*., K‐S *D*‐statistics) ranged from 0.03 to 0.84 (mean = 0.26) across the across the 264 K–S tests for all (*i.e*., both well‐surveyed and not‐well‐surveyed) grid cells surveyed since 1990. For well‐surveyed grid cells, *D*‐statistics were higher on average (0.31) and ranged from 0.06 to 0.82 across the 132 K–S tests. This variability in coverage indices suggests that coverage varied substantially among data sources, gradients, and taxonomic groups (Table 2). With regard to temporal coverage, GBIF records were more uniformly distributed (mean *D *=* *0.50 and 0.52 for all surveyed grid cells and well‐surveyed grid cells, respectively) compared to the BBS (*D *=* *0.73 and 0.73) and FFS records (*D *=* *0.81 and 0.82). Lower temporal bias in GBIF surveys compared to standardized surveys was likely a consequence of the longer time span from which GBIF records have been compiled (Fig. 3).

**Table 2:** Coverage indices along temporal, spatial, and environmental gradients for surveys derived from three open‐access databases representing ten taxonomic groups. Index values are *D‐*statistics from Kolmogorov–Smirnov goodness‐of‐fit tests and indicate strong or weak (low or high *D‐*statistics, respectively) congruence between survey datasets and the background environment. Index values are shown for all surveyed grid cells and well‐surveyed grid cells (based moderate or high thresholds). For the purpose of relative comparison, the mean *D‐*statistic across all 264 tests is 0.26

Grid cells	Taxon	Database	Temporal	Spatial^a^		Natural environment^a^			Anthropogenic land cover^a^			Climate change^a^	
			20‐year intervals	Lat.	Lon.	Elev.	MAT	MAP	Urban	Agriculture	Disturbed	∆MAP	∆MAT
All surveyed	Amphibians	GBIF	0.50	0.27	0.14	0.11	0.28	0.20	0.13	0.12	0.08	0.20	0.28
	Birds	GBIF	0.34	0.06	0.18	0.05	0.11	0.03	0.15	0.07	0.07	0.13	0.18
	Birds	BBS	0.73	0.05	0.17	0.12	0.06	0.16	0.13	0.08	0.11	0.07	0.10
	Bivalves	GBIF	0.61	0.45	0.62	0.53	0.47	0.60	0.33	0.27	0.31	0.20	0.48
	Crayfish	GBIF	0.63	0.25	0.52	0.59	0.28	0.50	0.41	0.23	0.33	0.27	0.21
	Fish	GBIF	0.58	0.27	0.32	0.30	0.33	0.38	0.26	0.19	0.24	0.08	0.25
	Fish	FFS	0.81	0.10	0.29	0.22	0.11	0.31	0.24	0.18	0.21	0.23	0.09
	Fungi	GBIF	0.54	0.07	0.16	0.05	0.09	0.17	0.06	0.18	0.15	0.09	0.18
	Insects	GBIF	0.47	0.17	0.08	0.11	0.15	0.07	0.12	0.06	0.04	0.11	0.07
	Mammals	GBIF	0.48	0.07	0.16	0.11	0.04	0.09	0.07	0.18	0.12	0.17	0.09
	Plants	GBIF	0.42	0.09	0.22	0.12	0.05	0.07	0.03	0.17	0.14	0.15	0.12
	Reptiles	GBIF	0.49	0.36	0.14	0.07	0.36	0.06	0.06	0.12	0.09	0.32	0.15
Well‐surveyed	Amphibians	GBIF	0.49	0.29	0.33	0.21	0.28	0.34	0.28	0.32	0.20	0.21	0.44
	Birds	GBIF	0.34	0.19	0.26	0.22	0.16	0.16	0.37	0.20	0.12	0.24	0.29
	Birds	BBS	0.73	0.06	0.18	0.13	0.06	0.18	0.14	0.09	0.12	0.08	0.09
	Bivalves	GBIF	0.75	0.66	0.73	0.66	0.68	0.84	0.40	0.32	0.34	0.30	0.62
	Crayfish	GBIF	n/a	n/a	n/a	n/a	n/a	n/a	n/a	n/a	n/a	n/a	n/a
	Fish	GBIF	0.67	0.48	0.49	0.36	0.50	0.54	0.31	0.28	0.31	0.16	0.36
	Fish	FFS	0.82	0.11	0.35	0.28	0.15	0.35	0.29	0.23	0.27	0.25	0.10
	Fungi	GBIF	0.70	0.22	0.43	0.31	0.14	0.17	0.17	0.43	0.39	0.25	0.21
	Insects	GBIF	0.51	0.13	0.29	0.10	0.12	0.10	0.10	0.21	0.13	0.16	0.19
	Mammals	GBIF	0.44	0.17	0.30	0.25	0.10	0.10	0.11	0.33	0.30	0.31	0.11
	Plants	GBIF	0.49	0.26	0.74	0.21	0.15	0.22	0.08	0.40	0.30	0.56	0.56
	Reptiles	GBIF	0.44	0.39	0.32	0.22	0.32	0.14	0.12	0.32	0.26	0.49	0.29

a Computed for contemporary time period (1990–2013).

Cumulative coverage indices (*i.e*., *D*‐statistics summed for the eleven spatial, environmental, and temporal gradients) suggested that the BBS bird and GBIF insect surveys had the best coverage, whereas GBIF surveys of bivalves and fish had the worst coverage (Fig. 4B). For most taxa, well‐surveyed grid cells exhibited worse cumulative coverage (upper left diagonal in Fig. 4B) than did all (*i.e*., both well‐surveyed and not‐well‐surveyed) surveyed grid cells. Exceptions included BBS birds, which contained well‐surveyed grid cells that exhibited similar coverage to all surveyed grid cells, and to a lesser degree FFS fish (1:1 line in Fig. 4B).

**Figure 5:**
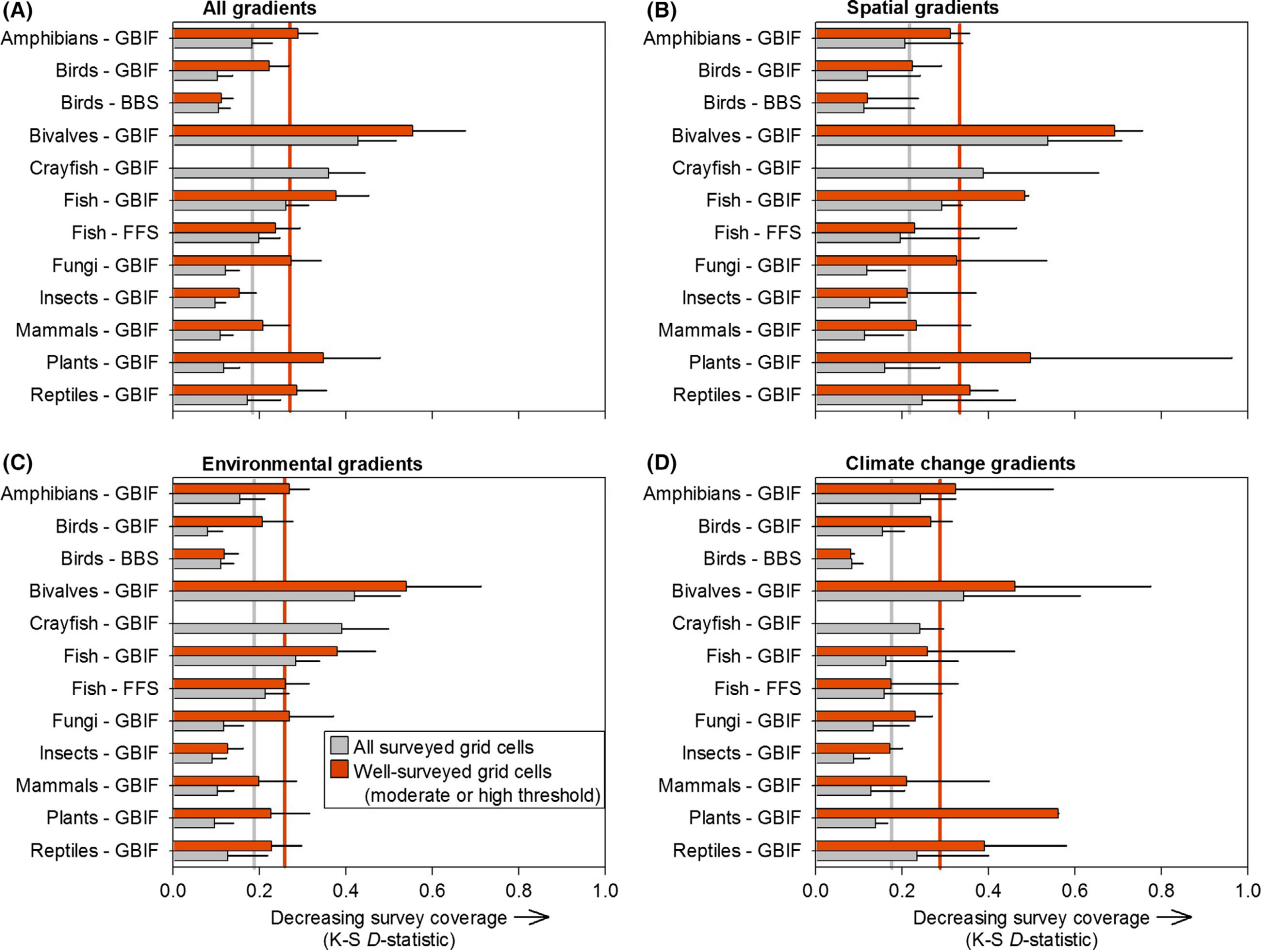
Coverage indices for each of twelve taxonomic survey datasets (eleven gradients pooled) averaged across (A) all eleven gradients, (B) two spatial gradients, (C) five contemporary environmental gradients (MAT, MAP, urban, agriculture, total disturbance), and (D) two climate change gradients (∆MAT, ∆MAP). Index values are *D‐*statistics from Kolmogorov–Smirnov goodness‐of‐fit, indicating strong or weak (low or high *D‐*statistics, respectively) congruence between survey datasets and the background environment. Vertical gray and red lines represent the mean of all twelve survey datasets for all surveyed grid cells and well‐surveyed grid cells, respectively.

Averaged coverage indices (*i.e*., *D‐*statistics) across all eleven temporal, spatial, and environmental gradients indicate that GBIF surveys of birds, insects, and mammals had the best coverage, whereas GBIF surveys of bivalves, crayfish, and fish were the most biased (Fig. 5A). For spatial gradients, the BBS bird surveys had the best coverage, followed by the GBIF insect surveys and the FFS fish surveys. By contrast, the GBIF bivalve, GBIF fish, and GBIF crayfish surveys had the worst spatial coverage (Fig. 5B). For environmental gradients, the BBS bird survey had the best coverage, followed by the GBIF insect and fungi surveys. By contrast, the GBIF surveys of bivalves, crayfish, and fish had the worst coverage (Fig. 5C). For forecasted gradients of climate change, the BBS bird surveys had the best coverage, followed by the GBIF insect surveys. By contrast, the GBIF surveys of bivalves, plants, and reptiles had the worst coverage along gradients of future climate change (Fig. 5D).

**Figure 6:**
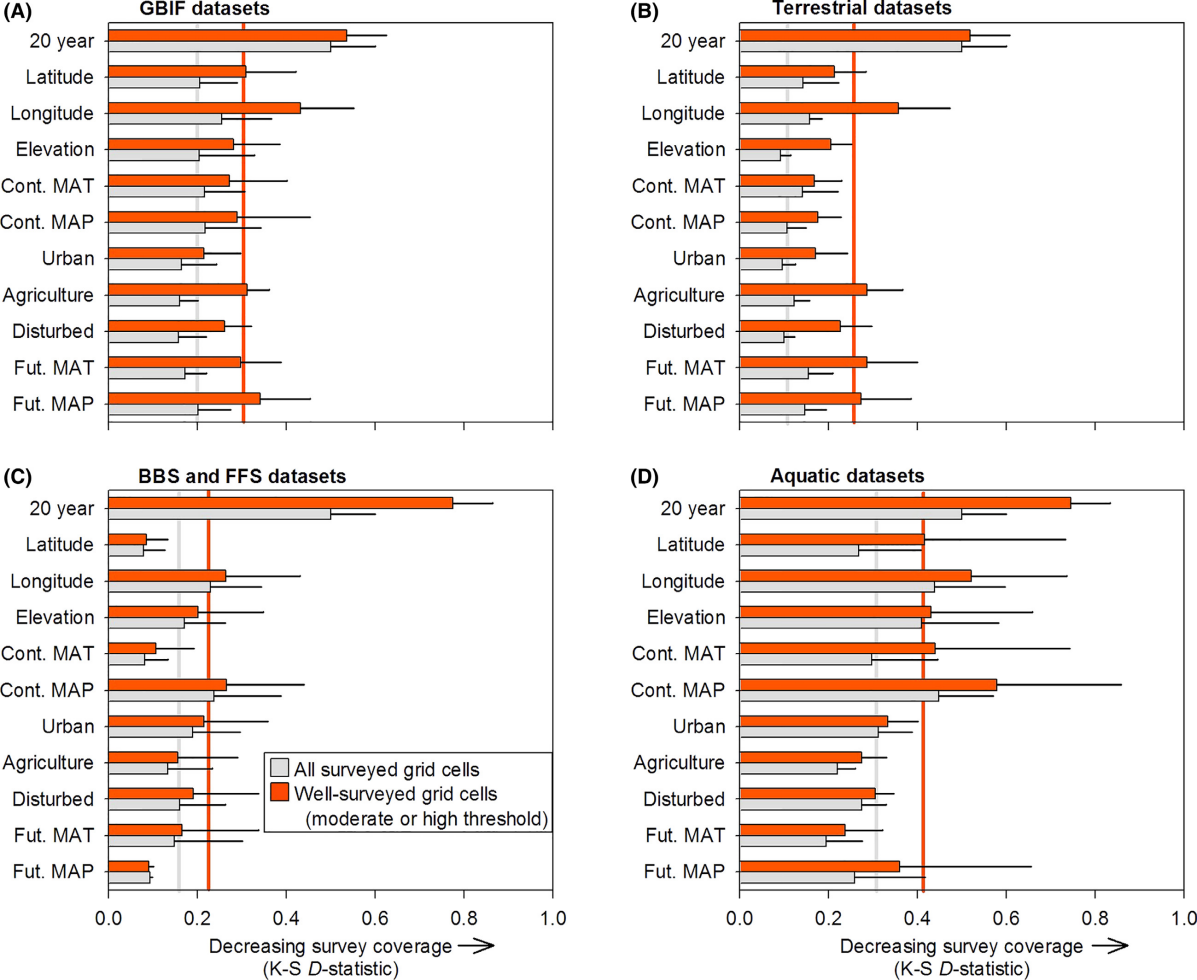
Coverage indices for each of eleven temporal, spatial, or environmental gradients (twelve taxonomic survey datasets pooled) averaged across (A) GBIF, (B) standardized (*i.e*., BBS and FFS), (C) terrestrial, and (D) aquatic datasets. Index values are *D‐*statistics from Kolmogorov–Smirnov goodness‐of‐fit, indicating strong or weak (low or high *D‐*statistics, respectively) congruence between survey datasets and the background environment. Vertical gray and red lines represent the mean of all eleven datasets for all surveyed grid cells and well‐surveyed grid cells, respectively.

Averaged coverage indices (*i.e*., *D‐*statistics) across all ten GBIF datasets indicate gradients of agricultural land cover, disturbed land cover, and urban land cover had the best coverage, whereas the temporal, longitudinal, and forecasted change in mean annual temperature gradients had the worst coverage (Fig. 6A). For standardized datasets (*i.e*., BBS and FFS), gradients of latitude, forecasted change in mean annual temperature, and contemporary mean annual temperature had the best coverage, whereas the temporal, longitudinal, and contemporary MAP gradients had the worst coverage (Fig. 6C). Terrestrial taxa (*i.e*., amphibians, birds, fungi, insects, mammals, plants, and reptiles) generally had better coverage than aquatic taxa (*i.e*., bivalves, crayfish, and fish) (Fig. 6B and D). For terrestrial taxa, gradients of contemporary mean annual temperature, contemporary mean annual precipitation, and urban land cover had the best coverage, whereas the temporal, longitudinal, and future MAT gradients had the worst coverage (Fig. 6B). For aquatic taxa, gradients of agricultural land cover, future MAP, and disturbed land cover had the worst coverage, whereas the temporal, contemporary MAP, and longitudinal gradients had the worst coverage (Fig. 6D).

Although *D‐*statistics summarized survey coverage along the entirety of a given gradient, the specific locations along that gradient that were over‐ or under‐represented were not characterized by these *D‐*statistics. A detailed description of every histogram here would be exhaustive, so we provide the raw histograms for all 264 dataset‐by‐taxon‐by‐gradient combinations as Supporting Information (Figs. S2.1–S2.10).

## Discussion

Open‐access biodiversity databases are essential to biodiversity research and conservation (Soberón and Peterson, 2004; Peterson et al. 2010); however, the efficacy of these databases depends on the completeness of species inventories and the coverage of surveys across dimensions of space, environment, and time (Kadmon et al. 2003; Hortal et al. 2008; Ladle and Hortal 2013). Many assessments have been completed for regions of the world including Central America, South America, the Iberian Peninsula, and western Africa (Hortal et al. 2007, 2008; Soberón et al. 2007; Sousa‐Baena et al. 2013; Idohou et al. 2015) as well as the entire globe (Meyer et al. 2015). Still, no study to our knowledge has evaluated completeness and/or coverage of open‐access biodiversity data for the United States. Our compilation of the Global Biodiversity Information Facility (GBIF), the North American Breeding Bird Survey (BBS), and federally administered freshwater fish surveys (FFS) yielded in excess of 13.6 million occurrence records distributed among more than 190,000 survey grid cells within the contiguous United States. By evaluating multiple datasets and taxonomic groups, simultaneously, our findings provide novel insights into the Wallacean shortfall (Lomolino and Lawrence 2004; Hortal et al. 2015). This comparative approach to biodiversity informatics provides a relative understanding of data needs for ten of the most abundant and diverse macro‐organism groups in the contiguous United States.

Open‐access biodiversity datasets differ in the type and origin of occurrence records they contain. For example, GBIF contains occurrence records that are often represented by individually vouchered museum specimens (Edwards et al. 2000; Yesson et al. 2007), whereas the BBS is a standardized whole‐assemblage surveying effort aimed at inventorying all breeding bird species along each survey route (Pardieck et al. 2014). The FFS is intermediate in that it contains standardized whole‐assemblage surveys, but there is variation in completeness stemming from the surveys being carried out with different survey methods of different government entities (Gilliom et al. 1995). Not surprisingly, the standardized surveys (BBS and FFS) produced a substantially higher proportion of well‐surveyed grid cells than GBIF‐derived surveys of birds and fishes, owing to the larger number of individual records (and accumulated species) per grid cell (Hortal et al. 2007). Despite providing a low proportion of well‐surveyed grid cells, GBIF still provided a sufficient number of well‐surveyed grid cells for most taxa because the database contains so many more independent occurrence records than the BBS and FFS. Across all ten GBIF taxa, the average number of well‐surveyed grid cells was 912 for the complete time period and 300 for the contemporary time period. Previous studies suggest that site‐by‐species matrices of this size are sufficient to produce accurate SDMs using presence–absence techniques or accurately model and map patterns in species richness (Lobo and Martín‐Piera 2002; Wisz et al. 2008). Thus, our evaluation of survey completeness demonstrates that the quantity and quality of data contained in all three datasets are suitable for biodiversity and SDM studies for most of the ten taxonomic groups.

Survey resolution is an important consideration that directly affected the completeness of species inventories (Hortal et al. 2006; Soberón et al. 2007). The size of grid cells we chose for the present study – approximately 100 km^2^ – is on the lower end of the size spectrum for studies of this type (*e.g*., Hortal et al. 2008; Yang et al., 2013; Sousa‐Baena et al. 2013). As such, most grid cells did not contain adequate densities of GBIF records to be classified as well‐surveyed. Nevertheless, those that did contain adequate densities of GBIF records offer richness and species occupancy information at a spatial resolution useful for biodiversity conservation and research (Rahbek 2005). One disadvantage of aggregating occurrence records by ~100 km^2^ grid cells is that finer‐grained spatial resolution of the systematic surveys is lost. This is because BBS routes and FFS stream reaches represent species inventories of areas that are smaller than the ~100 km^2^ grid cells. Given that most grid cells contained only a single BBS route or FFS reach, there was no advantage to aggregating occurrence records from these datasets by grid cells because information from multiple BBS routes or FFS reaches was not accumulated in a way that increases grid cell completeness. We conclude that aggregating occurrence records by grid cells is an effective technique for studies that use GBIF by itself or for studies that compile records from multiple databases, but not for studies using only the BBS or FFS databases.

The time period over which occurrence records were accumulated is another key factor that affected the completeness of species inventories. Our analysis revealed many more well‐surveyed grid cells for the 214‐year complete time period compared to the 24‐year contemporary time period. Although the time period over which occurrence records are aggregated will depend on the question being addressed (*e.g*., Rahel 2000; Pearman et al. 2008; Tingley and Beissinger 2009), these findings demonstrate that surveys derived from GBIF data are of sufficient quality and quantity for studies addressing contemporary or historical biodiversity of most taxonomic groups. It is also encouraging that GBIF records aggregated into 20‐year intervals yielded reasonably large numbers of well‐surveyed grid cells for several taxonomic groups going back to the late nineteenth century. Future efforts that identify historical time periods of high collection density could be used to optimize aggregation intervals, as opposed to the arbitrary 20‐year intervals used in the present evaluation, and likely increase the number of historically well‐surveyed grid cells (Hortal et al. 2008).

Many recent efforts have sought to characterize the Wallacean shortfall for individual taxonomic groups, particularly plants (*e.g*., Sousa‐Baena et al. 2013; Yang et al., 2014) and insects (*e.g*., Hortal et al. 2008; Beck et al. 2013). Whereas these single‐taxon studies are highly informative to conservation and research efforts within a given taxonomic group, comprehensive biodiversity conservation requires knowledge about data limitations of many taxonomic groups relative to one another (Funk et al. 2005; Meyer et al. 2015). Based on our simultaneous evaluation of ten taxonomic groups, it is apparent that the severity of the Wallacean shortfall varies substantially among taxonomic groups, an issue that has been described previously as the Linnean shortfall (Whittaker et al. 2005; Brito 2010). One notable trend is the lack of GBIF occurrence records for freshwater invertebrates, particularly crayfish and freshwater bivalves. Indeed, occurrence records for crayfish are so scare that no well‐surveyed grid cells were identified, even based on the low (*i.e*., least conservative) completeness thresholds. This is surprising given the imperilment of these taxa and the volume of research directed their way in recent years (Thorp and Covich 2009; Haag 2012; Ross 2013). Fungi were also poorly represented in GBIF relative to the other taxa, probably due to their cryptic life history and the relative paucity of research directed at documenting their distributions (Mueller et al. 2007). Overall, this quantitative evaluation thus provides an objective ranking (see Fig. 1) of primary biodiversity data needs for ten of the most abundant and diverse macro‐organism groups in the United States.

Another notable trend that became apparent from evaluating coverage of multiple taxonomic groups is the “taxonomist surveying bias”; that is, the tendency for collectors (both professional and recreational) to survey locations where the taxon of interest is most abundant or diverse (Sastre and Lobo 2009). For example, our analysis shows that the frequency of reptile surveys is highest in the desert southwest where reptiles are abundant, diverse, and frequently collected and studied. By contrast, reptile records are infrequent at higher latitudes and eastern longitudes, where reptiles still occur but are less abundant and diverse (Kiester 1971) and, presumably, collected and studied less frequently (Sastre and Lobo 2009). Similarly, freshwater fishes and bivalves are highly diverse and frequently studied in the southeastern United States and consequently have been collected frequently and vouchered in museums of this region (*e.g*., Tulane Museum of Natural History; Warren et al. 2000; Haag 2012). This pattern of geographic bias in survey coverage for freshwater fishes mirrors the findings of a recent assessment of global fish biodiversity data (Pelayo‐Villamil et al. 2015). Institutional participation in data compilation projects such as GBIF also influences coverage. This is apparent in the high density of surveys for particular taxonomic groups in some states but not in others. For example, the Kansas Biological Survey has made extensive collections of amphibians throughout the state of Kansas and has made these records electronically accessible via GBIF. This pattern is also evident for GBIF crayfish in Oklahoma and GBIF fish in North Carolina. Accessibility of sampling locations via road corridors and population centers is also a common driver of bias in survey coverage (Dennis and Hardy 1999; Kadmon et al. 2004). This source of coverage bias was not evident for any of the ten taxa, probably because the coarse survey resolution precluded our ability to detect biases in coverage along these finer resolution environmental characteristics.

Survey coverage varied among spatial, environmental, and temporal gradients. Not surprisingly, GBIF surveys exhibited higher spatial and environmental bias compared to the BBS and FFS, which represent systematic sampling efforts that are planned to be spatially and environmentally stratified (Gilliom et al. 1995; Pardieck et al. 2014). On the other hand, an advantage of museum record compilations such as GBIF is that the temporal distribution of records is typically longer and more uniform than systematic sampling efforts. Indeed, the first GBIF records were collected in the early 1800s, whereas the Breeding Bird Survey began in the 1960s and the FFS surveys span only the 1990s and 2000s. Another trend was consistently poorer coverage for aquatic taxa compared to terrestrial taxa. This may be a consequence of where aquatic habitats are most prevalent. For example, aquatic taxa surveys were overrepresented in wetter areas (*i.e*., higher mean annual precipitation; Fig. S2.5) of the eastern United States (Fig. S2.2). Nevertheless, unique and functionally diverse aquatic taxa persist in the arid southwest and other poorly covered regions (Pool and Olden 2012). Future efforts to fill in these aquatic biodiversity data gaps should therefore be a priority. Apart from these general trends, each taxon–gradient combination exhibited unique biases (Figs. S2.1–S2.10) that should be considered by collectors on a taxon‐specific basis when planning new data compilation and surveying efforts. Recent studies have highlighted that environmental biases vary in their effect on the performance of predictive models in other regions (Kadmon et al. 2003; Loiselle et al. 2008; Tessarolo et al. 2014). To what degree the environmental biases documented in the current study would affect predictive models remains unknown and should be a future objective of biodiversity informatics in the United States.

Effective biodiversity conservation starts with researchers and conservationists having access to biodiversity surveys of sufficient completeness and coverage (Reichman et al. 2011). Evaluations like the one we present provide a quantitative and comprehensive prioritization scheme to facilitate efficient improvements to existing databases, such as GBIF. Another essential goal of such prioritization schemes should be to produce future data coverages that enable the study of long‐term biodiversity responses to anthropogenic environmental change (*e.g*., Jiguet et al. 2010). Such an approach should involve the identification of areas that presently are undersurveyed and are expected to undergo anthropogenic environmental change over the next 50–100 years. Such foresight in data collection by this generation of scientists can provide complete and unbiased “before” data for BACI‐designed natural experiments conducted by scientists 50–100 years into the future after environmental change has occurred. Indeed, our evaluation suggests that climate change gradients are among the most poorly covered environmental gradients. Lastly, descriptive evaluations of completeness and coverage like the one we present should be viewed as an iterative process. Investigators will need to periodically reevaluate completeness and coverage as new occurrence records are added to open‐access databases. Such periodic reevaluations will need to incorporate additional coverage information, as new environmental data layers become available or as existing environmental data layers change as a consequence of climate and land cover change.

## Conflict of Interest

None declared.

## Supporting information


**Figure S1.** Spatial and environmental variables summarized at the resolution of 0.1° by 0.1° grid cells (N = 83,545) used in coverage analysis.

**Figure S2.1**. Distribution of occurrence records along a latitudinal spatial gradient.
**Figure S2.2**. Distribution of occurrence records along a longitudinal spatial gradient.
**Figure S2.3**. Distribution of occurrence records along a gradient of elevation.
**Figure S2.4**. Distribution of occurrence records along a gradient of mean annual temperature.
**Figure S2.5**. Distribution of occurrence records along a gradient of mean annual precipitation.
**Figure S2.6**. Distribution of occurrence records along a gradient of urban land cover.
**Figure S2.7**. Distribution of occurrence records along a gradient of agricultural land cover.
**Figure S2.8**. Distribution of occurrence records along a gradient of disturbed (urban + agricultural) land cover.
**Figure S2.9**. Distribution of occurrence records along a gradient of change (future – present) in mean annual temperature.
**Figure S2.10**. Distribution of occurrence records along a gradient of change (future – present) in mean annual precipitation.

## References

[ece32225-bib-0001] Bahn, V. , and B. J. McGill . 2007 Can niche‐based distribution models outperform spatial interpolation? Glob. Ecol. Biogeogr. 16:733–742.

[ece32225-bib-0002] Bates, B. C. , Z. W. Kundzewicz , S. Wu , and J. Palutikof . 2008 Climate Change and Water. Technical Paper of the Intergovernmental Panel on Climate Change, IPCC Secretariat, Geneva.

[ece32225-bib-0003] Beck, J. , L. Ballesteros‐Mejia , P. Nagel , and I. J. Kitching . 2013 Online solutions and the ‘Wallacean shortfall’: what does GBIF contribute to our knowledge of species’ ranges? Divers. Distrib. 19:1043–1050.

[ece32225-bib-0004] Bennett, E. M. , S. R. Carpenter , and N. F. Caraco . 2001 Human impact on erodable phosphorus and eutrophication: a global perspective increasing accumulation of phosphorus in soil threatens rivers, lakes, and coastal oceans with eutrophication. Bioscience 51:227–234.

[ece32225-bib-0005] Brito, D. 2010 Overcoming the Linnean shortfall: data deficiency and biological survey priorities. Basic Appl. Ecol., 11, 709–713.

[ece32225-bib-0006] Broennimann, O. , U.A. Treier , H. Müller‐Schärer , W. Thuiller , A.T. Peterson , and A. Guisan . 2007 Evidence of climatic niche shift during biological invasion. Ecol. Lett., 10, 701–709. 17594425 10.1111/j.1461-0248.2007.01060.x

[ece32225-bib-0007] Brown, J. H. 1995 Macroecology. University of Chicago Press, Chicago.

[ece32225-bib-0500] Colwell, R. K. , and J. A. Coddington . 1994 Estimating terrestrial biodiversity through extrapolation. Philos. T. Roy. Soc. B. 345:101‐118. 10.1098/rstb.1994.0091 7972351

[ece32225-bib-0008] Chao, A. 1987 Estimating the population size for capture‐recapture data with unequal catchability. Biometrics 43:783–791. 3427163

[ece32225-bib-0009] Chao, A. , and L. Jost . 2012 Coverage‐based rarefaction and extrapolation: standardizing samples by completeness rather than size. Ecology 93:2533–2547. 23431585 10.1890/11-1952.1

[ece32225-bib-0010] Dennis, R. L. , and P. B. Hardy . 1999 Targeting squares for survey: predicting species richness and incidence of species for a butterfly atlas. Glob. Ecol. Biogeogr. 8:443–454.

[ece32225-bib-0011] Dennis, R. L. H. , and C. D. Thomas . 2000 Bias in butterfly distribution maps: the influence of hot spots and recorder's home range. J. Insect Conserv. 4:73–77.

[ece32225-bib-0012] Domínguez‐Domínguez, O. , A. R. Martínez‐Meyer , E. Zambrano , L. De , and G. Perez‐Ponce . 2006 Using ecological‐niche modeling as a conservation tool for freshwater species: live‐bearing fishes in central Mexico. Conserv. Biol. 20:1730–1739. 17181808 10.1111/j.1523-1739.2006.00588.x

[ece32225-bib-0014] Edwards, J. L. , M. A. Lane , and E. S. Nielsen . 2000 Interoperability of biodiversity databases: biodiversity information on every desktop. Science 289:2312–2314. 11009409 10.1126/science.289.5488.2312

[ece32225-bib-0015] Esselman, P. C. , D. M. Infante , L. Wang , D. Wu , A. R. Cooper , and W. W. Taylor . 2011 An index of cumulative disturbance to river fish habitats of the conterminous United States from landscape anthropogenic activities. Ecol. Rest. 29:133–151.

[ece32225-bib-0016] Funk, V. A. , K. S. Richardson , and S. Ferrier . 2005 Survey‐gap analysis in expeditionary research: where do we go from here? Biol. J. Linn. Soc. 85:549–567.

[ece32225-bib-0017] García‐Roselló, E. , C. Guisande , A. Manjarrés‐Hernández , J. González‐Dacosta , J. Heine , P. Pelayo‐Villamil , et al. 2015 Can we derive macroecological patterns from primary Global Biodiversity Information Facility data? Glob. Ecol. Biogeogr. 24:335–347.

[ece32225-bib-0018] Gilliom, R. J. , W. M. Alley , and M. E. Gurtz . 1995 Design of the national water‐quality assessment program: occurrence and distribution of water‐quality conditions. U.S. Geological Survey, Reston, Virginia.

[ece32225-bib-0019] Giovanelli, J. G. , C. F. Haddad , and J. Alexandrino . 2008 Predicting the potential distribution of the alien invasive American bullfrog (*Lithobates catesbeianus*) in Brazil. Biol. Invasions 10:585–590.

[ece32225-bib-0020] Girvetz, E. H. , C. Zganjar , G. T. Raber , E. P. Maurer , P. Kareiva , and J. J. Lawler . 2009 Applied climate‐change analysis: the climate wizard tool. PLoS ONE 4:e8320. 20016827 10.1371/journal.pone.0008320 PMC2790086

[ece32225-bib-0021] Guisan, A. , and W. Thuiller . 2005 Predicting species distribution: Offering more than simple habitat models. Ecol. Lett. 8:993–1009. 10.1111/j.1461-0248.2005.00792.x 34517687

[ece32225-bib-0022] Haag, W. R. 2012 North American freshwater mussels: natural history, ecology, and conservation. Cambridge University Press, London, UK.

[ece32225-bib-0023] Herlihy, A. T. , R. M. Hughes , and J. Sifneos . 2006 Landscape clusters based on fish assemblages in the conterminous USA and their relationship to existing landscape classifications Pp. 87–112 *in* Hughes R. M., Wang L. and Seelbach P. W., eds. Landscape influences on stream habitats and biological assemblages. American Fisheries Society Symposium, Bethesda.

[ece32225-bib-0024] Hijmans, R. J. , S. E. Cameron , J. L. Parra , P. G. Jones , and A. Jarvis . 2005 Very high resolution interpolated climate surfaces for global land areas. Int. J. Climatol. 25:1965–1978.

[ece32225-bib-0025] Hortal, J. , and J. M. Lobo . 2005 An ED‐based protocol for the optimal sampling of biodiversity. Biodivers. Conserv. 14:2913–2947.

[ece32225-bib-0026] Hortal, J. , P. A. Borges , and C. Gaspar . 2006 Evaluating the performance of species richness estimators: sensitivity to sample grain size. J. Anim. Ecol. 75:274–287. 16903065 10.1111/j.1365-2656.2006.01048.x

[ece32225-bib-0027] Hortal, J. , J. M. Lobo , and A. Jimenez‐Valverde . 2007 Limitations of Biodiversity Databases: Case Study on Seed‐Plant Diversity in Tenerife, Canary Islands. Conserv. Biol. 21:853–863. 17531062 10.1111/j.1523-1739.2007.00686.x

[ece32225-bib-0028] Hortal, J. , A. Jiménez‐Valverde , J. F. Gómez , J. M. Lobo , and A. Baselga . 2008 Historical bias in biodiversity inventories affects the observed environmental niche of the species. Oikos 117:847–858.

[ece32225-bib-0029] Hortal, J. , F. de Bello , J. A. F. Diniz‐Filho , T. M. Lewinsohn , J. M. Lobo , and R. J. Ladle . 2015 Seven shortfalls that beset large‐scale knowledge of biodiversity. Annu. Rev. Ecol. Evol. Syst. 46:523–549.

[ece32225-bib-0030] Idohou, R. , A. H. Arino , A. E. Assogbadjo , and R. Glele . 2015 Knowledge of diversity of wild palms (Arecaceae) in the republic of Benin: finding gaps in the national inventory by combining field and digital accessible knowledge. Biodiver. Informat. 10:45–55.

[ece32225-bib-0031] Jiguet, F. , R. D. Gregory , V. Devictor , R. E. Green , P. Vorisek , A. Van Strien , et al. 2010 Population trends of European common birds are predicted by characteristics of their climatic niche. Glob. Change Biol. 16:497–505.

[ece32225-bib-0032] Kadmon, R. , O. Farber , and A. Danin . 2003 A systematic analysis of factors affecting the performance of climatic envelope models. Ecol. Appl. 13:853–867.

[ece32225-bib-0033] Kadmon, R. , O. Farber , and A. Danin . 2004 Effect of roadside bias on the accuracy of predictive maps produced by bioclimatic models. Ecol. Appl. 14:401–413.

[ece32225-bib-0034] Kiester, A. R. 1971 Species density of North American amphibians and reptiles. Syst. Zool. 20:127–137.

[ece32225-bib-0035] Kozak, K. H. , and J. J. Wiens . 2006 Does niche conservatism promote speciation? A case study in North American salamanders. Evolution 60:2604–2621. 17263120

[ece32225-bib-0036] Ladle, R. , and J. Hortal . 2013 Mapping species distributions: living with uncertainty. Frontiers of Biogeography 5:9.

[ece32225-bib-0037] Lobo, J. M. 2008 Database records as a surrogate for sampling effort provide higher species richness estimations. Biodivers. Conserv. 17:873–881.

[ece32225-bib-0038] Lobo, J. M. , and F. Martín‐Piera . 2002 Searching for a predictive model for species richness of Iberian dung beetle based on spatial and environmental variables. Conserv. Biol. 16:158–173. 10.1046/j.1523-1739.2002.00211.x 35701967

[ece32225-bib-0039] Loiselle, B. A. , P. M. Jørgensen , T. Consiglio , I. Jiménez , J. G. Blake , L. G. Lohmann , et al. 2008 Predicting species distributions from herbarium collections: does climate bias in collection sampling influence model outcomes? J. Biogeogr. 35:105–116.

[ece32225-bib-0040] Lomolino, M.V.H. , and R. Lawrence . 2004 Frontiers of biogeography: new directions in the geography of nature (No. 578.09 F7).

[ece32225-bib-0041] Meyer, C. , H. Kreft , R. Guralnick , and W. Jetz . 2015 Global priorities for an effective information basis of biodiversity distributions. Nat. Commun. 6: doi:10.1038/ncomms9221. 10.1038/ncomms9221 PMC4569846 26348291

[ece32225-bib-0042] Mims, M. C. , and J. D. Olden . 2012 Life history theory predicts fish assemblage response to hydrologic regimes. Ecology 93:35–45. 22486085 10.1890/11-0370.1

[ece32225-bib-0043] Mitchell, A. L. , and J. H. Knouft . 2009 Non‐native fishes and native species diversity in freshwater fish assemblages across the United States. Biol. Invasions 11:1441–1450.

[ece32225-bib-0044] Mueller, G. M. , J. P. Schmit , P. R. Leacock , B. Buyck , J. Cifuentes , D. E. Desjardin , et al. 2007 Global diversity and distribution of macrofungi. Biodivers. Conserv. 16:37–48.

[ece32225-bib-0045] Newbold, T. , L. N. Hudson , S. L. Hill , S. Contu , I. Lysenko , R. A. Senior , et al. 2015 Global effects of land use on local terrestrial biodiversity. Nature 520:45–50. 25832402 10.1038/nature14324

[ece32225-bib-0046] Oksanen, J. , R. Kindt , P. Legendre , B. O'Hara , M. H. H. Stevens , M. J. Oksanen , and MASS . 2007 The vegan package. *Community ecology package*, 10.

[ece32225-bib-0047] Pardieck, K.L. , D.J. Ziolkowski , and M‐A.R. Hudson . 2014 North American Breeding Bird Survey Dataset 1966 ‐ 2013, version 2013.0. U.S. Geological Survey, Patuxent Wildlife Research Center. Available at www.pwrc.usgs.gov/BBS/RawData/.

[ece32225-bib-0048] Pearman, P. B. , A. Guisan , O. Broennimann , and C. F. Randin . 2008 Niche dynamics in space and time. Trends Ecol. Evol. 23:149–158. 18289716 10.1016/j.tree.2007.11.005

[ece32225-bib-0049] Pelayo‐Villamil, P. , C. Guisande , R. P. Vari , A. Manjarrés‐Hernández , E. García‐Roselló , J. González‐Dacosta , et al. 2015 Global diversity patterns of freshwater fishes–potential victims of their own success. Divers. Distrib. 21:345–356.

[ece32225-bib-0050] Peterson, A. T. , M. Papeş , and M. Eaton . 2007 Transferability and model evaluation in ecological niche modeling: a comparison of GARP and Maxent. Ecography 30:550–560.

[ece32225-bib-0051] Peterson, A. T. , S. Knapp , R. Guralnick , J. Soberón , and M. T. Holder . 2010 The big questions for biodiversity informatics. Syst. Biodivers. 8:159–168.

[ece32225-bib-0052] Phillips, S. J. , R. Anderson , and R. Schapire . 2006 Maximum entropy modeling of species geographic distributions. Ecol. Model. 190:231–259.

[ece32225-bib-0053] Pineda, E. , and J. M. Lobo . 2009 Assessing the accuracy of species distribution models to predict amphibian species richness patterns. J. Anim. Ecol. 78:182–190. 18771504 10.1111/j.1365-2656.2008.01471.x

[ece32225-bib-0054] Poff, N. L. , J. D. Olden , D. M. Merritt , and D. M. Pepin . 2007 Homogenization of regional river dynamics by dams and global biodiversity implications. Proc. Natl Acad. Sci. 104:5732–5737. 17360379 10.1073/pnas.0609812104 PMC1851560

[ece32225-bib-0055] Pool, T. K. , and J. D. Olden . 2012 Taxonomic and functional homogenization of an endemic desert fish fauna. Divers. Distrib. 18:366–376.

[ece32225-bib-0056] R Core Team (2014). R: a language and environment for statistical computing. R Foundation for Statistical Computing, Vienna, Austria Available at http://www.R-project.org/.

[ece32225-bib-0057] Rahbek, C. 2005 The role of spatial scale and the perception of large‐scale species‐richness patterns. Ecol. Lett. 8:224–239.

[ece32225-bib-0058] Rahel, F. J. 2000 Homogenization of fish faunas across the United States. Science 288:854–856. 10797007 10.1126/science.288.5467.854

[ece32225-bib-0059] Reichman, O. J. , M. B. Jones , and M. P. Schildhauer . 2011 Challenges and opportunities of open data in ecology. Science 331:703–705. 21311007 10.1126/science.1197962

[ece32225-bib-0060] Ross, S. T. 2013 Ecology of North American freshwater fishes. University of California Press, Oakland, CA, USA.

[ece32225-bib-0061] Sastre, P. , and J. M. Lobo . 2009 Taxonomist survey biases and the unveiling of biodiversity patterns. Biol. Conserv. 142:462–467.

[ece32225-bib-0062] Soberón, J. , and T. Peterson . 2004 Biodiversity informatics: managing and applying primary biodiversity data. Phil. Trans. R. Soc. B: Biol. Sci. 359:689–698. 10.1098/rstb.2003.1439 PMC1693343 15253354

[ece32225-bib-0063] Soberón, J. , R. Jiménez , J. Golubov , and P. Koleff . 2007 Assessing completeness of biodiversity databases at different spatial scales. Ecography 30:152–160.

[ece32225-bib-0064] Sousa‐Baena, M. S. , L. C. Garcia , and A. T. Peterson . 2013 Completeness of digital accessible knowledge of the plants of Brazil and priorities for survey and inventory. Divers. Distrib. 20:369–381.

[ece32225-bib-0065] Tessarolo, G. , T. F. Rangel , M. B. Araújo , and J. Hortal . 2014 Uncertainty associated with survey design in Species Distribution Models. Divers. Distrib. 20:1258–1269.

[ece32225-bib-0066] Thorp J.H., and Covich A.P., eds. 2009 Ecology and classification of North American freshwater invertebrates. Academic Press, Cambridge, MA, USA.

[ece32225-bib-0067] Tingley, M. W. , and S. R. Beissinger . 2009 Detecting range shifts from historical species occurrences: new perspectives on old data. Trends Ecol. Evol. 24:625–633. 19683829 10.1016/j.tree.2009.05.009

[ece32225-bib-0068] Turner, M. G. , R. H. Gardner , and R. V. O'Neill . 2001 Landscape ecology in theory and practice: pattern and process. Springer Science & Business Media, New York.

[ece32225-bib-0069] Vavrek, M. J. 2011 fossil: palaeoecological and palaeogeographical analysis tools. Palaeontologia Electronica, 14:1T. Available at http://palaeo-electronica.org/2011_1/238/index.html

[ece32225-bib-0070] Warren, M. L. , B. M. Burr , S. J. Walsh , H. L. Bart , R. C. Cashner , D. A. Etnier , et al. 2000 Diversity, distribution, and conservation status of the native freshwater fishes of the southern United States. Fisheries 25:7–31.

[ece32225-bib-0071] Whittaker, R. J. , M. B. Araújo , P. Jepson , R. J. Ladle , J. E. Watson , and K. J. Willis . 2005 Conservation biogeography: assessment and prospect. Divers. Distrib. 11:3–23.

[ece32225-bib-0072] Wisz, M. S. , R. J. Hijmans , J. Li , A. T. Peterson , C. H. Graham , and A. Guisan . 2008 Effects of sample size on the performance of species distribution models. Divers. Distrib. 14:763–773.

[ece32225-bib-0073] Yang, W. , K. Ma , and J. Kreft . 2013 Geographic sampling bias in a large distributional database and its effects on species richness–environment models. J. Biogeogr. 40:1415–1426.

[ece32225-bib-0074] Yesson, C. , P. W. Brewer , T. Sutton , N. Caithness , J. S. Pahwa , M. Burgess , et al. 2007 How global is the global biodiversity information facility? PLoS One 2:e1124. 17987112 10.1371/journal.pone.0001124 PMC2043490

