# Supplementary figures and images for: Filling in the GAPS: evaluating completeness and coverage of open‐access biodiversity databases in the United States
Source: Ecol Evol. 2016 Jun 12;6(14):4654–69. doi: 10.1002/ece3.2225 (PMC4979697; doi:10.1002/ece3.2225)

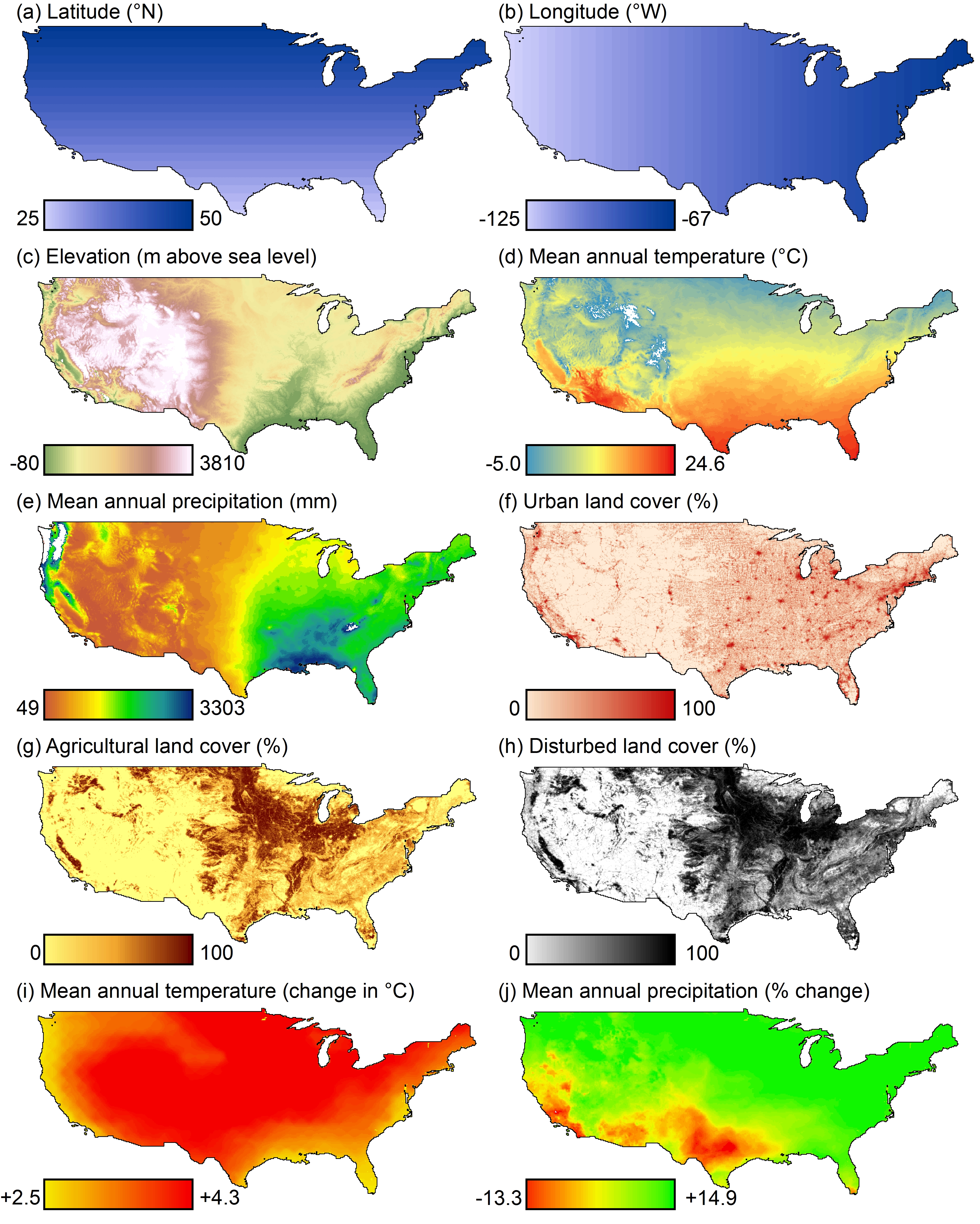

Supplement: Supplementary file 1 — Figure S1. Spatial and environmental variables summarized at the resolution of 0.1° by 0.1° grid cells (N = 83,545) used in coverage analysis. [file ECE3-6-4654-s001.jpg]

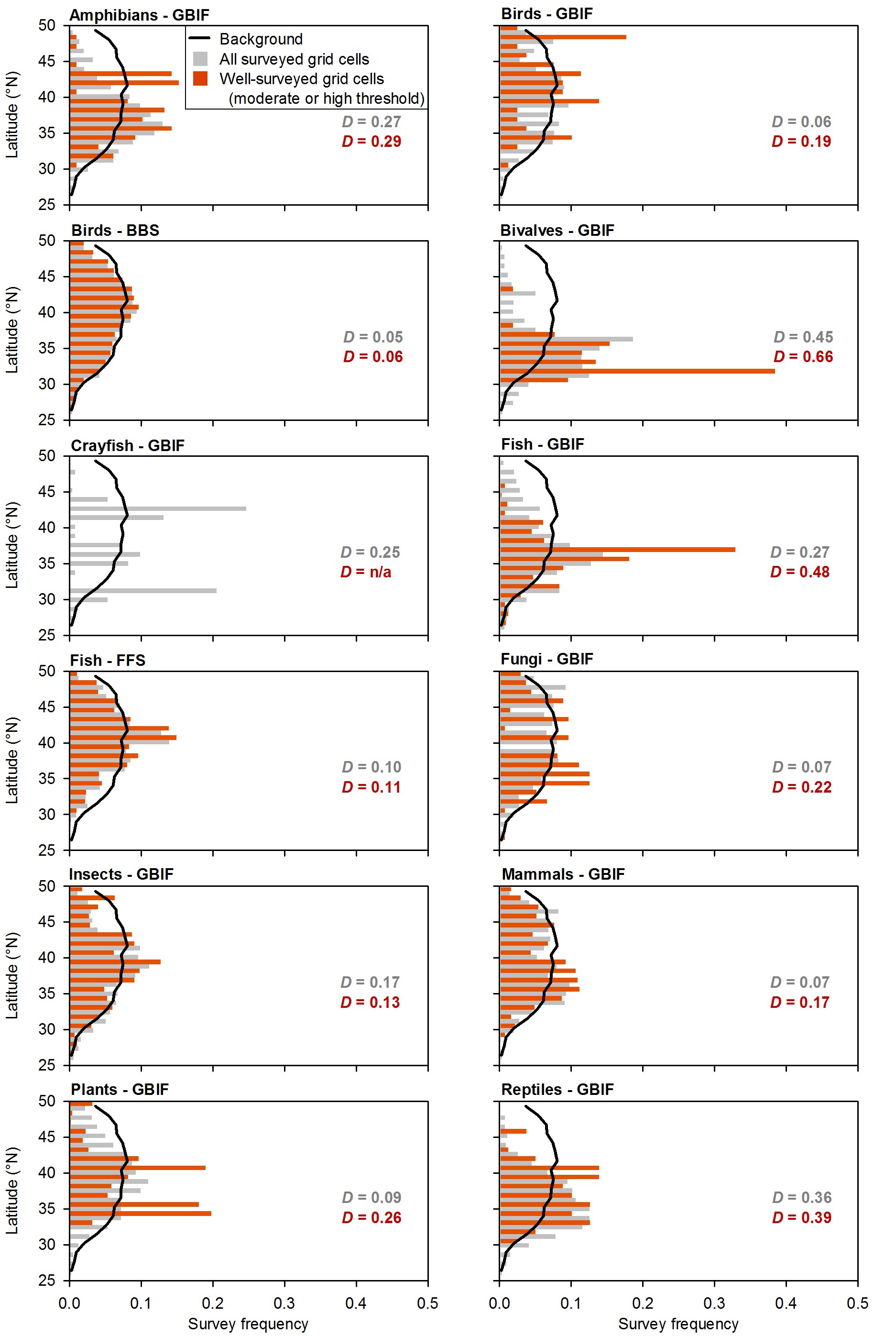

Supplement: Supplementary file 2 — Figure S2.1. Distribution of occurrence records along a latitudinal spatial gradient. Figure S2.2. Distribution of occurrence records along a longitudinal spatial gradient. Figure S2.3. Distribution of occurrence records along a gradient of elevation. Figure S2.4. Distribution of occurrence records along a gradient of mean annual temperature. Figure S2.5. Distribution of occurrence records along a gradient of mean annual precipitation. Figure S2.6. Distribution of occurrence records along a gradient of urban land cover. Figure S2.7. Distribution of occurrence records along a gradient of agricultural land cover. Figure S2.8. Distribution of occurrence records along a gradient of disturbed (urban + agricultural) land cover. Figure S2.9. Distribution of occurrence records along a gradient of change (future – present) in mean annual temperature. Figure S2.10. Distribution of occurrence records along a gradient of change (future – present) in mean annual precipitation. [file ECE3-6-4654-s002.zip › ece32225-sup-0002-FigS2.1.JPG]

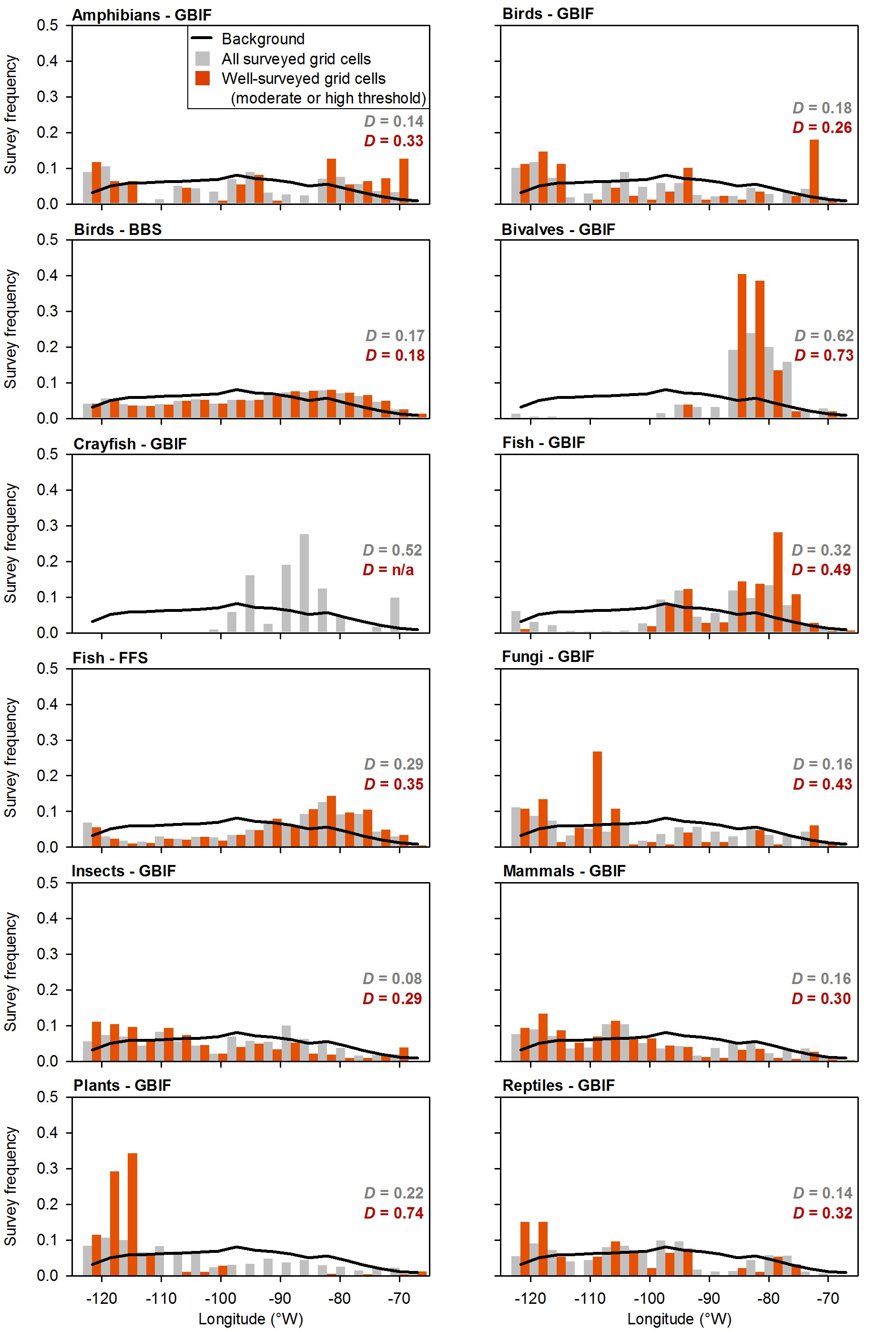

Supplement: Supplementary file 2 — Figure S2.1. Distribution of occurrence records along a latitudinal spatial gradient. Figure S2.2. Distribution of occurrence records along a longitudinal spatial gradient. Figure S2.3. Distribution of occurrence records along a gradient of elevation. Figure S2.4. Distribution of occurrence records along a gradient of mean annual temperature. Figure S2.5. Distribution of occurrence records along a gradient of mean annual precipitation. Figure S2.6. Distribution of occurrence records along a gradient of urban land cover. Figure S2.7. Distribution of occurrence records along a gradient of agricultural land cover. Figure S2.8. Distribution of occurrence records along a gradient of disturbed (urban + agricultural) land cover. Figure S2.9. Distribution of occurrence records along a gradient of change (future – present) in mean annual temperature. Figure S2.10. Distribution of occurrence records along a gradient of change (future – present) in mean annual precipitation. [file ECE3-6-4654-s002.zip › ece32225-sup-0003-FigS2.2.JPG]

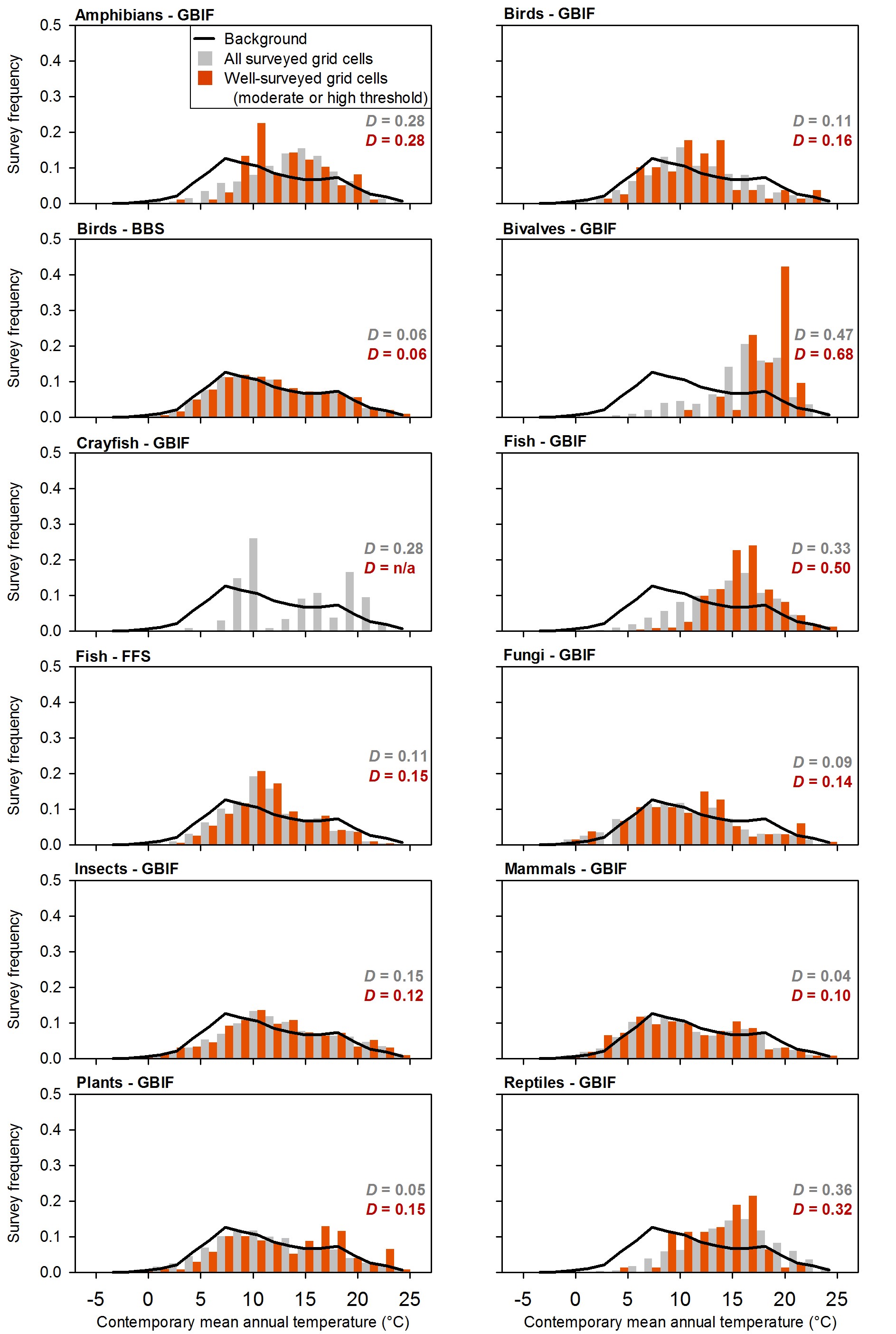

Supplement: Supplementary file 2 — Figure S2.1. Distribution of occurrence records along a latitudinal spatial gradient. Figure S2.2. Distribution of occurrence records along a longitudinal spatial gradient. Figure S2.3. Distribution of occurrence records along a gradient of elevation. Figure S2.4. Distribution of occurrence records along a gradient of mean annual temperature. Figure S2.5. Distribution of occurrence records along a gradient of mean annual precipitation. Figure S2.6. Distribution of occurrence records along a gradient of urban land cover. Figure S2.7. Distribution of occurrence records along a gradient of agricultural land cover. Figure S2.8. Distribution of occurrence records along a gradient of disturbed (urban + agricultural) land cover. Figure S2.9. Distribution of occurrence records along a gradient of change (future – present) in mean annual temperature. Figure S2.10. Distribution of occurrence records along a gradient of change (future – present) in mean annual precipitation. [file ECE3-6-4654-s002.zip › ece32225-sup-0003-FigS2.3.JPG]

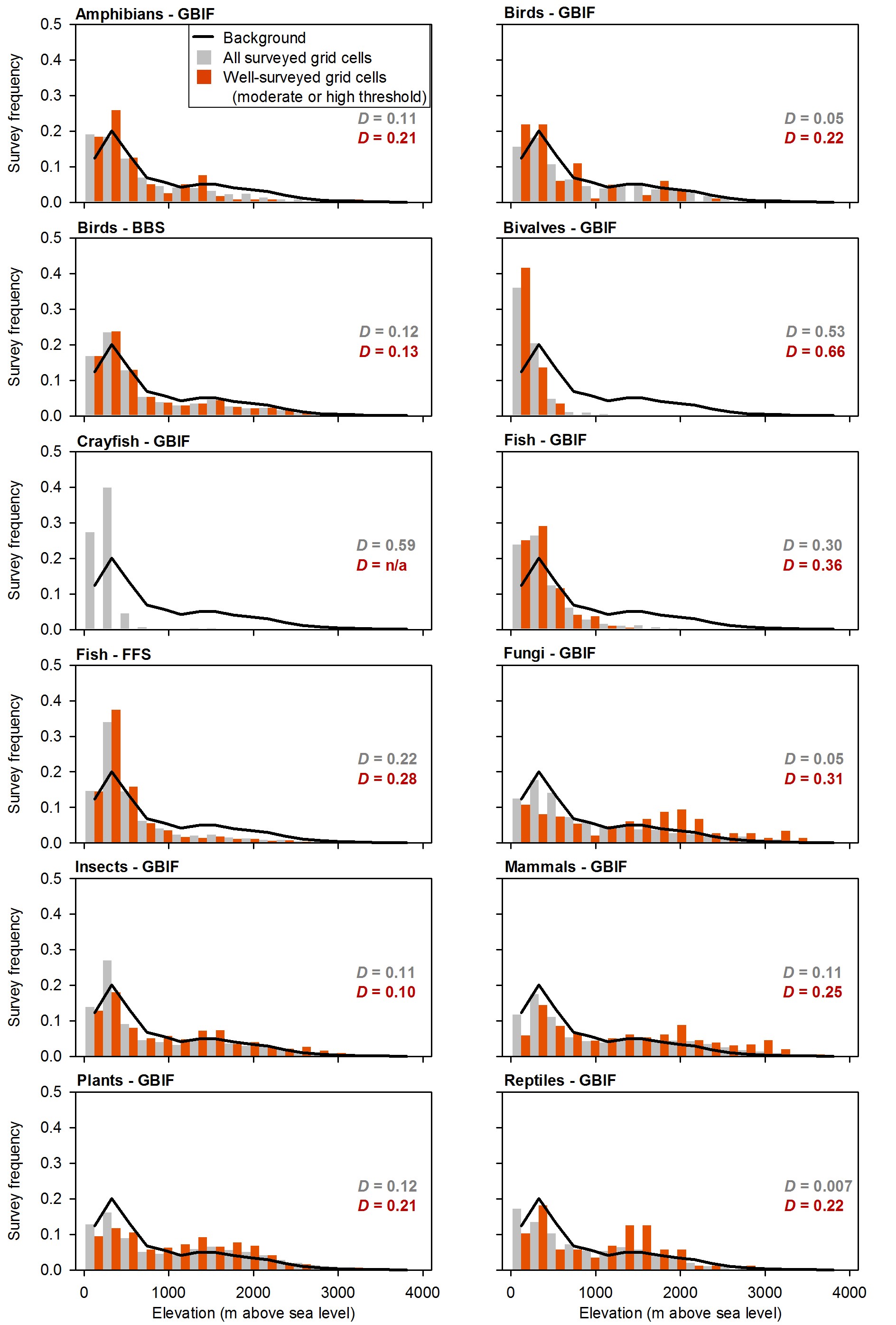

Supplement: Supplementary file 2 — Figure S2.1. Distribution of occurrence records along a latitudinal spatial gradient. Figure S2.2. Distribution of occurrence records along a longitudinal spatial gradient. Figure S2.3. Distribution of occurrence records along a gradient of elevation. Figure S2.4. Distribution of occurrence records along a gradient of mean annual temperature. Figure S2.5. Distribution of occurrence records along a gradient of mean annual precipitation. Figure S2.6. Distribution of occurrence records along a gradient of urban land cover. Figure S2.7. Distribution of occurrence records along a gradient of agricultural land cover. Figure S2.8. Distribution of occurrence records along a gradient of disturbed (urban + agricultural) land cover. Figure S2.9. Distribution of occurrence records along a gradient of change (future – present) in mean annual temperature. Figure S2.10. Distribution of occurrence records along a gradient of change (future – present) in mean annual precipitation. [file ECE3-6-4654-s002.zip › ece32225-sup-0004-FigS2.4.JPG]

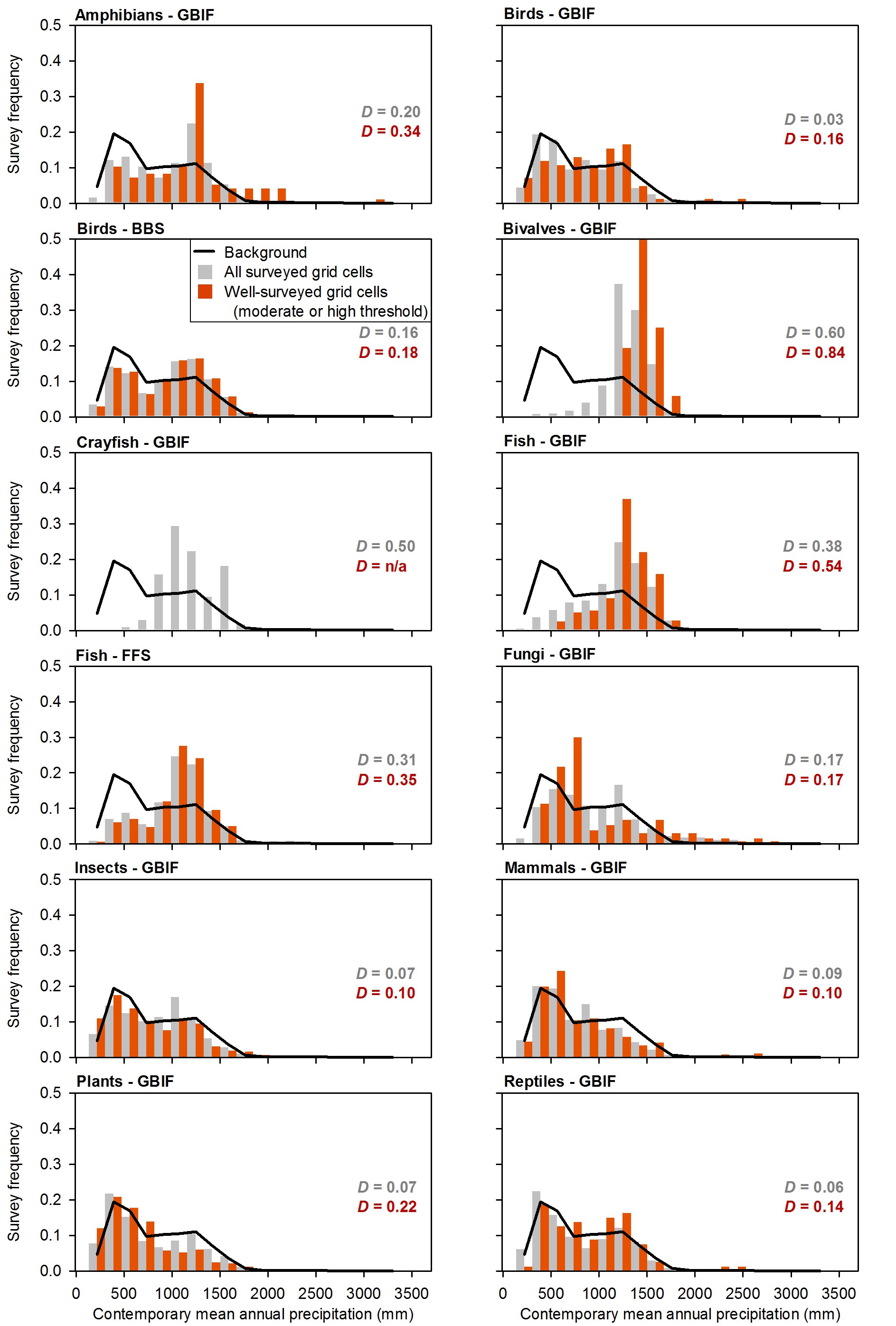

Supplement: Supplementary file 2 — Figure S2.1. Distribution of occurrence records along a latitudinal spatial gradient. Figure S2.2. Distribution of occurrence records along a longitudinal spatial gradient. Figure S2.3. Distribution of occurrence records along a gradient of elevation. Figure S2.4. Distribution of occurrence records along a gradient of mean annual temperature. Figure S2.5. Distribution of occurrence records along a gradient of mean annual precipitation. Figure S2.6. Distribution of occurrence records along a gradient of urban land cover. Figure S2.7. Distribution of occurrence records along a gradient of agricultural land cover. Figure S2.8. Distribution of occurrence records along a gradient of disturbed (urban + agricultural) land cover. Figure S2.9. Distribution of occurrence records along a gradient of change (future – present) in mean annual temperature. Figure S2.10. Distribution of occurrence records along a gradient of change (future – present) in mean annual precipitation. [file ECE3-6-4654-s002.zip › ece32225-sup-0005-FigS2.5.JPG]

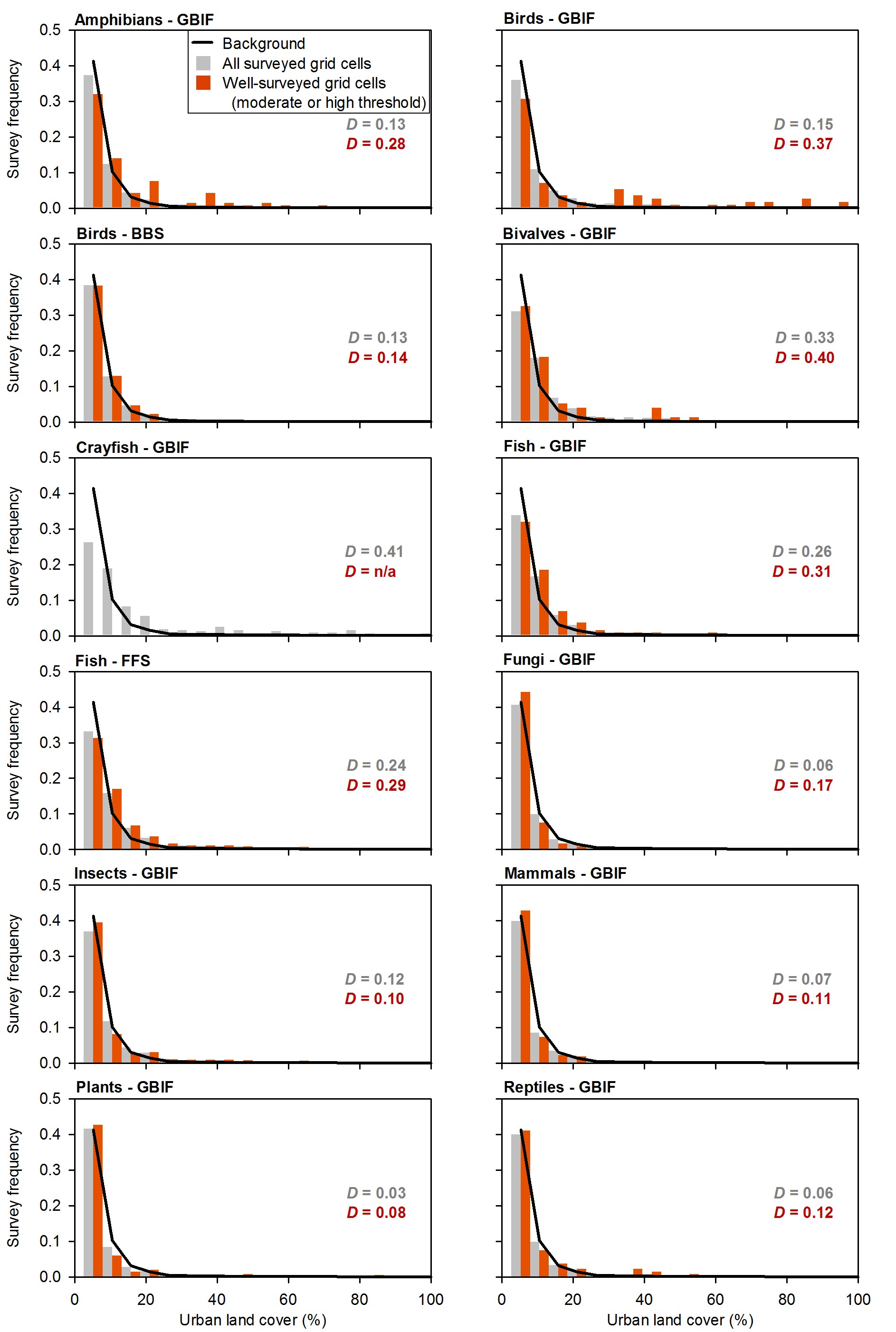

Supplement: Supplementary file 2 — Figure S2.1. Distribution of occurrence records along a latitudinal spatial gradient. Figure S2.2. Distribution of occurrence records along a longitudinal spatial gradient. Figure S2.3. Distribution of occurrence records along a gradient of elevation. Figure S2.4. Distribution of occurrence records along a gradient of mean annual temperature. Figure S2.5. Distribution of occurrence records along a gradient of mean annual precipitation. Figure S2.6. Distribution of occurrence records along a gradient of urban land cover. Figure S2.7. Distribution of occurrence records along a gradient of agricultural land cover. Figure S2.8. Distribution of occurrence records along a gradient of disturbed (urban + agricultural) land cover. Figure S2.9. Distribution of occurrence records along a gradient of change (future – present) in mean annual temperature. Figure S2.10. Distribution of occurrence records along a gradient of change (future – present) in mean annual precipitation. [file ECE3-6-4654-s002.zip › ece32225-sup-0006-FigS2.6.JPG]

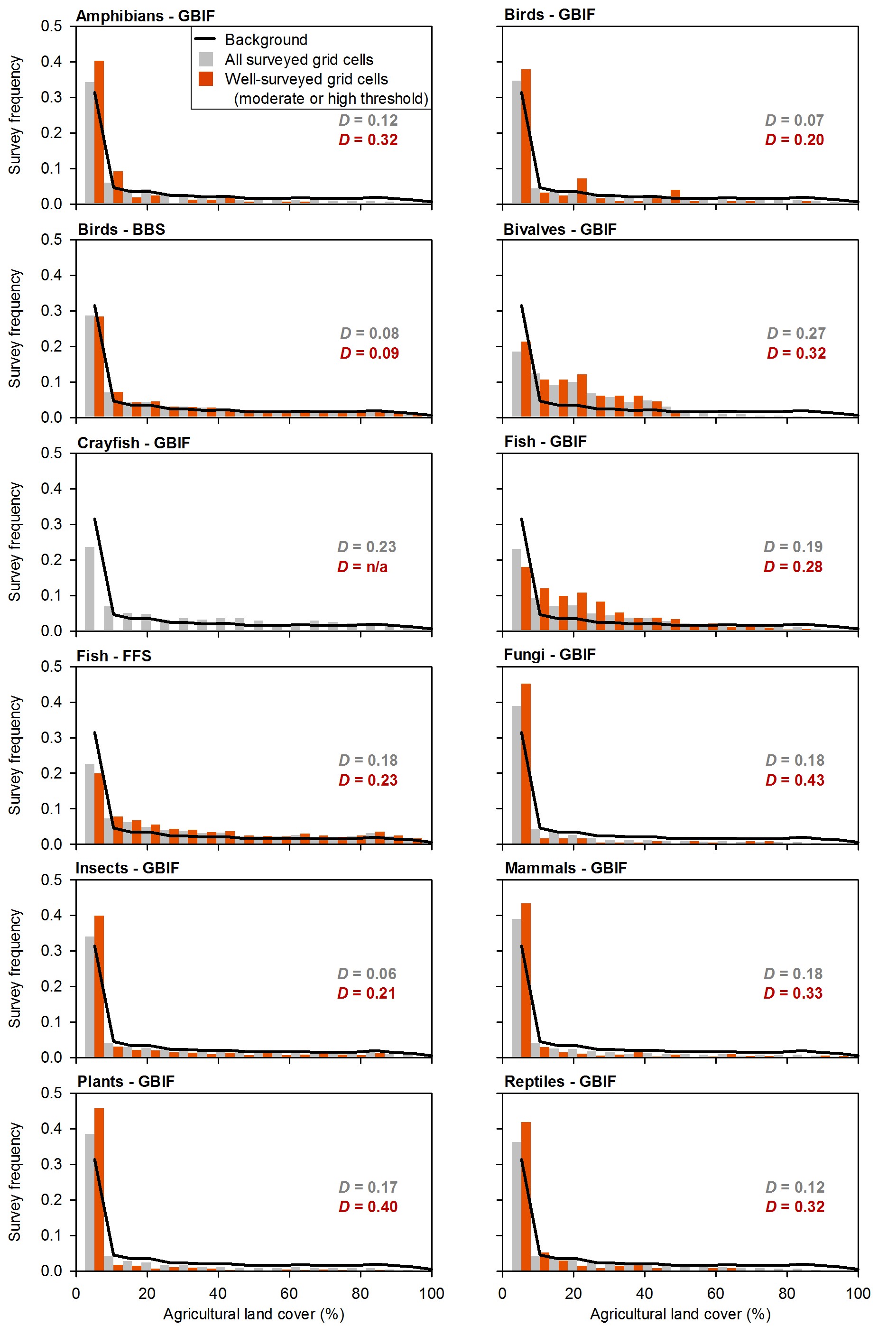

Supplement: Supplementary file 2 — Figure S2.1. Distribution of occurrence records along a latitudinal spatial gradient. Figure S2.2. Distribution of occurrence records along a longitudinal spatial gradient. Figure S2.3. Distribution of occurrence records along a gradient of elevation. Figure S2.4. Distribution of occurrence records along a gradient of mean annual temperature. Figure S2.5. Distribution of occurrence records along a gradient of mean annual precipitation. Figure S2.6. Distribution of occurrence records along a gradient of urban land cover. Figure S2.7. Distribution of occurrence records along a gradient of agricultural land cover. Figure S2.8. Distribution of occurrence records along a gradient of disturbed (urban + agricultural) land cover. Figure S2.9. Distribution of occurrence records along a gradient of change (future – present) in mean annual temperature. Figure S2.10. Distribution of occurrence records along a gradient of change (future – present) in mean annual precipitation. [file ECE3-6-4654-s002.zip › ece32225-sup-0007-FigS2.7.JPG]

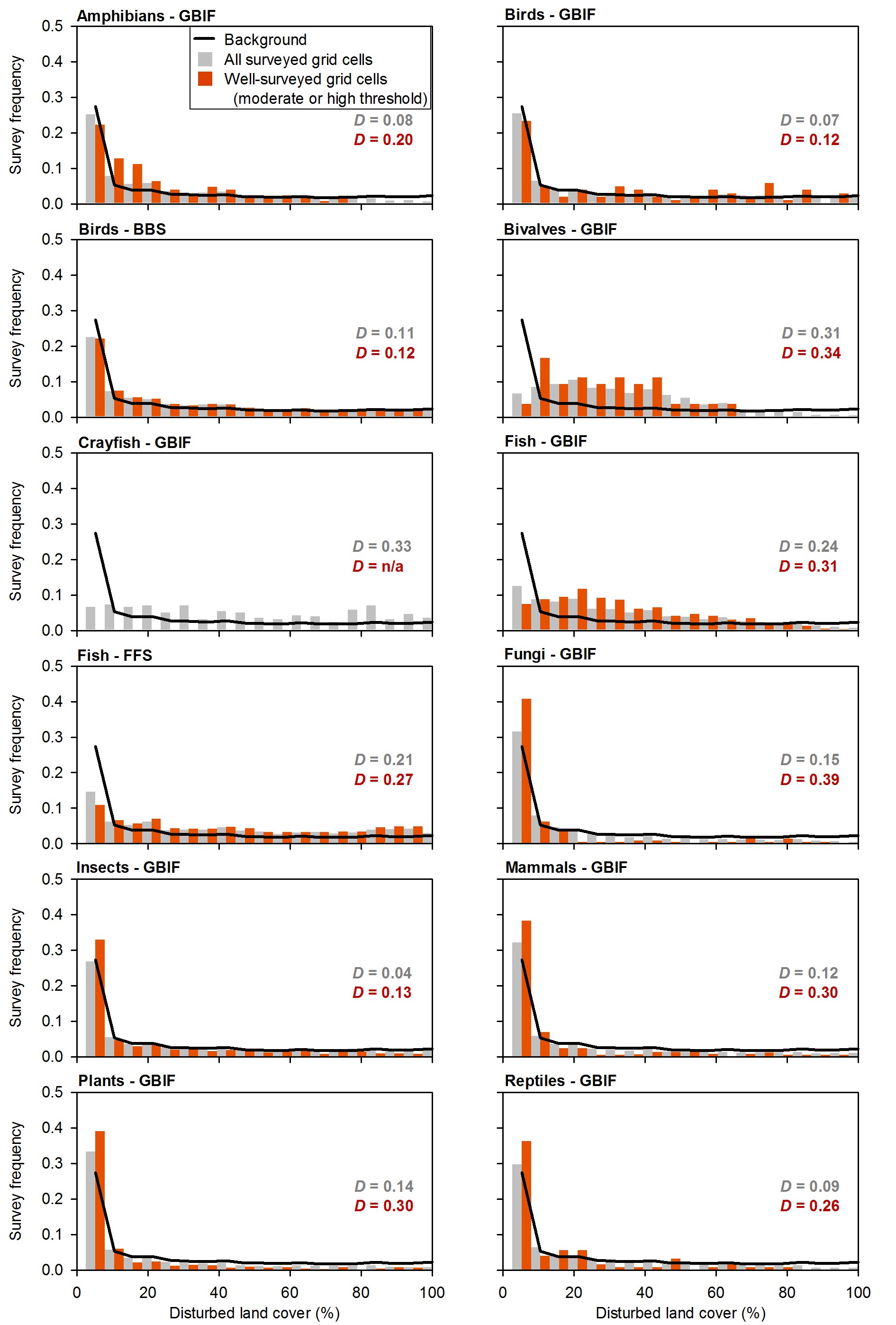

Supplement: Supplementary file 2 — Figure S2.1. Distribution of occurrence records along a latitudinal spatial gradient. Figure S2.2. Distribution of occurrence records along a longitudinal spatial gradient. Figure S2.3. Distribution of occurrence records along a gradient of elevation. Figure S2.4. Distribution of occurrence records along a gradient of mean annual temperature. Figure S2.5. Distribution of occurrence records along a gradient of mean annual precipitation. Figure S2.6. Distribution of occurrence records along a gradient of urban land cover. Figure S2.7. Distribution of occurrence records along a gradient of agricultural land cover. Figure S2.8. Distribution of occurrence records along a gradient of disturbed (urban + agricultural) land cover. Figure S2.9. Distribution of occurrence records along a gradient of change (future – present) in mean annual temperature. Figure S2.10. Distribution of occurrence records along a gradient of change (future – present) in mean annual precipitation. [file ECE3-6-4654-s002.zip › ece32225-sup-0008-FigS2.8.JPG]

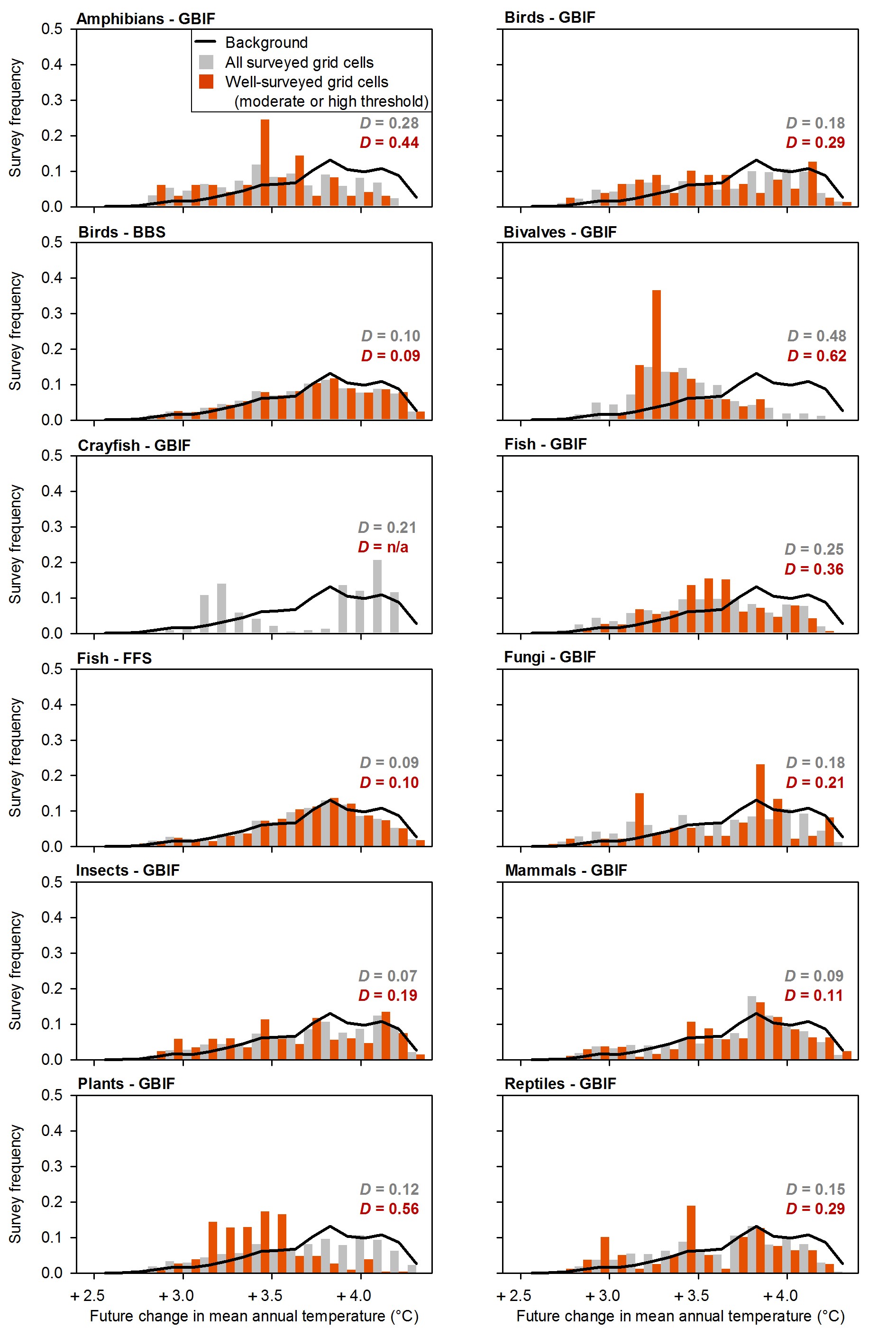

Supplement: Supplementary file 2 — Figure S2.1. Distribution of occurrence records along a latitudinal spatial gradient. Figure S2.2. Distribution of occurrence records along a longitudinal spatial gradient. Figure S2.3. Distribution of occurrence records along a gradient of elevation. Figure S2.4. Distribution of occurrence records along a gradient of mean annual temperature. Figure S2.5. Distribution of occurrence records along a gradient of mean annual precipitation. Figure S2.6. Distribution of occurrence records along a gradient of urban land cover. Figure S2.7. Distribution of occurrence records along a gradient of agricultural land cover. Figure S2.8. Distribution of occurrence records along a gradient of disturbed (urban + agricultural) land cover. Figure S2.9. Distribution of occurrence records along a gradient of change (future – present) in mean annual temperature. Figure S2.10. Distribution of occurrence records along a gradient of change (future – present) in mean annual precipitation. [file ECE3-6-4654-s002.zip › ece32225-sup-0009-FigS2.9.JPG]

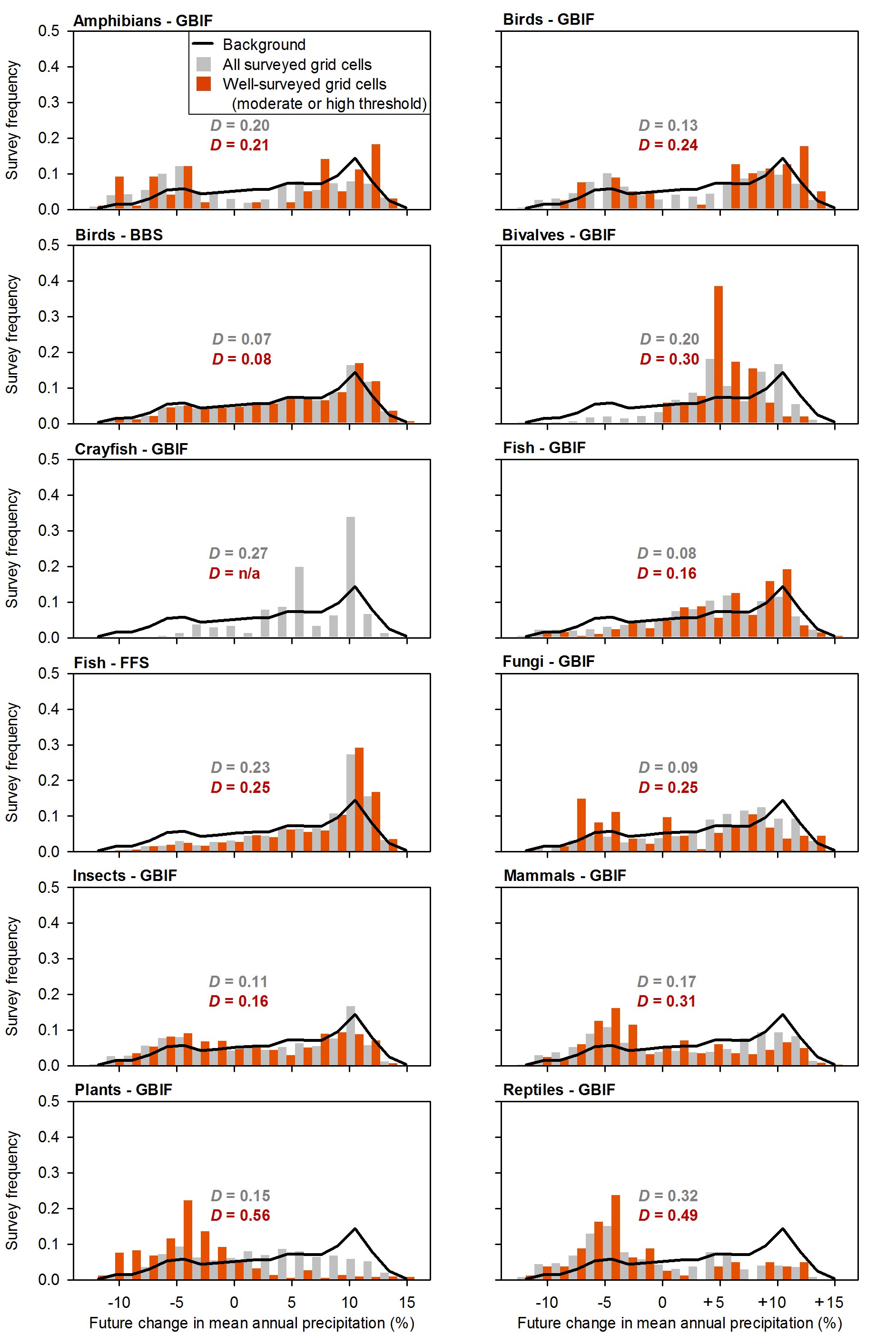

Supplement: Supplementary file 2 — Figure S2.1. Distribution of occurrence records along a latitudinal spatial gradient. Figure S2.2. Distribution of occurrence records along a longitudinal spatial gradient. Figure S2.3. Distribution of occurrence records along a gradient of elevation. Figure S2.4. Distribution of occurrence records along a gradient of mean annual temperature. Figure S2.5. Distribution of occurrence records along a gradient of mean annual precipitation. Figure S2.6. Distribution of occurrence records along a gradient of urban land cover. Figure S2.7. Distribution of occurrence records along a gradient of agricultural land cover. Figure S2.8. Distribution of occurrence records along a gradient of disturbed (urban + agricultural) land cover. Figure S2.9. Distribution of occurrence records along a gradient of change (future – present) in mean annual temperature. Figure S2.10. Distribution of occurrence records along a gradient of change (future – present) in mean annual precipitation. [file ECE3-6-4654-s002.zip › ece32225-sup-0010-FigS2.10.JPG]
